# Binary Mixture Droplet Evaporation on Microstructured Decorated Surfaces and the Mixed Stick–Slip Modes

**DOI:** 10.1021/acs.langmuir.3c00914

**Published:** 2023-06-05

**Authors:** Khaloud Moosa Al Balushi, Gail Duursma, Prashant Valluri, Khellil Sefiane, Daniel Orejon

**Affiliations:** † School of Engineering, Institute for Multiscale Thermofluids, The University of Edinburgh, Edinburgh EH9 3FD, Scotland, UK; ‡ College of Engineering and Technology, The University of Technology and Applied Sciences, Suhar 311, Oman; § International Institute for Carbon-Neutral Energy Research (WPI-I2CNER), Kyushu University, 744 Motooka, Nishi-ku, Fukuoka 819-0395, Japan

## Abstract

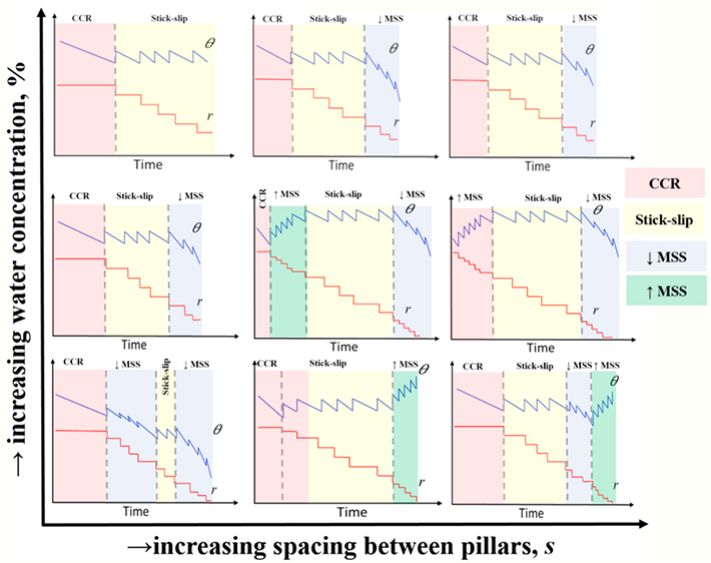

The interactions between liquid droplets and solid surfaces during wetting and phase change are important to many applications and are related to the physicochemical properties of the substrate and the fluid. In this work, we investigate experimentally the evaporation of pure water, pure ethanol, and their binary mixture droplets, accessing a wide range of surface tensions, on hydrophobic micro-pillared surfaces varying the spacing between the pillars. Results show that on structured surfaces, droplets evaporate following three classical evaporative behaviors: constant contact radius/pinning, stick–slip, or mixed mode. In addition, we report two further droplet evaporation modes, which are a mixed stick–slip mode where the contact angle increases while the contact radius decreases in a stick–slip fashion and a mixed stick–slip mode where both the contact angle and the contact radius decrease in a stick–slip fashion. We name these evaporation modes not yet reported in the literature as the increasing and decreasing contact angle mixed stick–slip modes, respectively. The former ensues because the fluid surface tension increases as the most volatile fluid evaporates coupled to the presence of structures, whereas the latter is due to the presence of structures for either fluid. The duration of each evaporation mode is dissimilar and depends on the surface tension and on the spacing between structures. Pure water yields longer initial pinning times on all surfaces before stick–slip ensues, whereas for binary mixtures and pure ethanol, initial pinning ensues mainly on short spacing structures due to the different wetting regimes displayed. Meanwhile, mixed stick–slip modes ensue mainly for high ethanol concentrations and/or pure ethanol independent of the solid fraction and for low ethanol concentrations on large spacing. Contact line jumps, changes in contact angle and pinning forces are also presented and discussed. This investigation provides guidelines for tailoring the evaporation of a wide range of surface tension fluids on structured surfaces for inkjet printing, DNA patterning, or microfluidics applications.

## Introduction

Although wetting and evaporation of liquid droplets appear to be simple phenomena, they are topics of great interest and relevant to many scientific advancements as well as research development, which relates to a wide range of industrial, agricultural, biological, and biomedical applications.^1,2^ Understanding and controlling the droplet contact line dynamics during wetting and evaporation phase change are crucial to expanding and improving common daily practices such as inkjet printing,^3,4^ spray cooling,^5^ DNA microarray fabrication,^6,7^ and coating technology,^8^ amongst others.

Droplet evaporation behavior on smooth solid substrates depends strongly on the wettability of the surfaces,^9−11^ the nature of the liquid used,^12,13^ and on the surrounding ambient.^14,15^ Traditionally, three main distinctive evaporation modes are reported in the literature: constant contact radius or pinning mode (CCR), constant contact angle mode (CCA), and mixed mode.^9,16,17^ On smooth hydrophilic surfaces, evaporation occurs initially in the CCR mode until a certain evaporation time and then it transitions into the mixed mode;^7^ if particles are added to the droplets, then evaporation ensues mainly in the CCR mode.^9,18^ In contrast, on smooth hydrophobic surfaces, droplets evaporate in the CCA mode during most of the droplet lifetime and in mixed mode at the very end of the evaporation with only a short CCR mode ensuing during the very first instants of the evaporation.^9^ Nevertheless, if particles are added to the droplet, then a droplet evaporates following an additional evaporation mode called the stick–slip mode.^9^ In this evaporation mode, local pinning of the contact line takes place while the contact angle decreases to account for evaporation, until a certain point at which the droplet contact line jumps/recedes with an associated increase in the droplet contact angle.^9,19^

The use of pure fluids has been widely studied and addressed in the literature; nonetheless, these pure fluids provide rather limited specific values of thermophysical properties, including surface tension. For binary mixtures, several works address wetting and evaporation on smooth surfaces.^12,13,20−25^ Sefiane *et al*.^20^ investigated ethanol–water binary mixture droplet evaporation at ambient pressure on PTFE surfaces. They concluded that for pure liquids and high water concentration mixtures, the evaporation process ensues in the CCA mode with a monotonic decrease in the contact radius during most of the droplet lifetime, while in the case of the binary mixtures with a lower water concentration, droplets evaporate following three different stages. In these binary mixtures, a mode of evaporation different from the CCA or CCR modes is observed in which the contact angle increases while the radius decreases as the most volatile and lowest surface tension fluid evaporates.^13,15,26^ The first stage of the evaporation of binary mixtures is governed by the evaporation of the most volatile fluid and the last stage by the less volatile fluid, while the intermediate stage shows different quantiative behavior depending on the ethanol–water concentration and initial wetting states.^20^ More recently, the increase in contact angle coupled with the decrease in the contact radius was also reported during the evaporation of pure ethanol droplets in humid air as a consequence of the adsorption-absorption and/or condensation of water vapor onto the surface, which modifies the droplet surface tension locally.^14,15^

Additionally, in the presence of structures, the wettability and surface structures strongly influence wetting, spreading, and the final shape of the droplet,^27−29^ which in turn play an important role in the static and the dynamics of the triple contact line during wetting and evaporation, *i.e.*, the evaporative modes.^17,30^ For pure fluids, the presence of structures prevents the occurrence of the CCA mode of evaporation in favor of either the CCR or the stick–slip modes as the additional structural sites present locations for the droplet contact line to pin.^30^ McHale *et al*. showed experimentally that pure water droplets initially evaporate in the CCR mode followed by the stick–slip mode on SU-8 textured surfaces with apparent contact angles above 140°.^31^ More recently, Chen *et al*. found out that water droplets exhibit three distinctive evaporation modes on hydrophobic structured surfaces, namely, CCR, CCA, and mixed modes.^32^ They further showed, experimentally, that the contact line dynamics of evaporating droplets can be controlled by changing the geometric arrangement of the substrate, *i.e.*, the pillar-to-pillar spacing.^32^ Xu *et al*. also noticed these three classical evaporation modes; CCR, CCA, and mixed mode, when using hydrophobic micro-pillared surfaces.^17^ Their study clearly shows that the evaporation modes depend highly on the morphology of the structures. When increasing the spacing between pillars, a longer duration of the CCA mode is observed when compared to the CCR mode. Consequently, the duration of each evaporation mode depends highly on the nature of the surface and the liquid used,^10,11,17,25,33^ and its control is crucial to many applications such as spray cooling and biosensors.^10^

In addition to the surface structure and fluid type, Dash and Garimella^10^ investigated the effect of pure water droplets on a superhydrophobic structured surface and found that by increasing the droplet volume, the evaporation mode can vary accordingly. Xu *et al*.^17^ also investigated the evaporation of pure water droplets on smooth and on four different micro-pillared surfaces having different spacing between pillars, *i.e.*, different solid fractions. Results further supported the similar distinctive evaporation modes to those already reported in the literature on rough surfaces, where droplets begin evaporating in the CCR mode, then transition to the CCA mode, and vanish with the mixed mode. They proposed that as the spacing between pillars increases, the duration of the CCR decreases, and the CCA mode duration increases with similar behavior to that on the smooth hydrophobic counterpart, while the duration of the mixed mode increases as spacing decreases.^17^ Presumably, distinctive stick–slip behavior must ensue during their reported CCA owing to the presence of structures underneath the evaporating droplets, which was not reported as per the low spatial and temporal resolution of their data.^17^ We highlight that most of the works on structured surfaces reported evaporation in the CCA mode and overlooked providing thorough details and analysis on the occurrence of the stick–slip phenomenon during their evaporation, as per the lack of temporal and spatial resolution of the measurements.

Only recently, the evaporation of binary mixtures on structured surfaces has received dedicated attention;^25,33−35^ nonetheless, there is still a lack of complete and beneficial understanding of the topic. Using binary mixtures on structured surfaces has a significant effect on wetting^27^ and evaporation^26^ characteristics. Feng *et al.* reported on the evaporation of squared and octagon low ethanol–water concentration droplets (below 30%) on micropyramid cavity substrates looking at the contact angle from two different azimuthal directions with differences within 12°.^36^ Droplets were found to evaporate in the CCR mode for most of the droplet lifetime, *i.e.*, from 40 to 60% of the time, with no qualitative apparent differences in the evaporation regimes displayed and their duration when looking at the droplet from different azimuthal directions. On micropyramid substrates and in the presence of surfactants, droplets displaying geometrical features such as a square or octagon remained pinned during most of the droplet lifetime.^37^ Chiang and Lu made use of water–methanol binary mixtures, at two different low methanol mole fractions, on superhydrophobic copper surfaces decorated with high-aspect-ratio nanostructured and on smooth ones for comparison.^25^ Low methanol mole fraction binary mixture droplets on nanostructured surfaces were found to evaporate in the CCR mode followed by the CCA mode and ending with the mixed mode, while no stick–slip mode was observed. Evaporation behaviors resembled those of pure water as per the rather low methanol concentrations studied. These findings are in agreement with the work of Yu *et al*.,^35^ who used ethanol–water binary mixtures at three different concentrations on a single PDMS microstructured surface configuration. By increasing the ethanol concentration of the liquid or by using pure ethanol, the evaporation behavior ensues following two distinctive evaporation modes instead of three. Evaporation occurs in the CCA mode for almost 80% of the initial evaporation lifetime, and droplets then vanish in the mixed mode without stick–slip. He *et al*. also investigated the full evaporation process of binary mixtures with a high ethanol concentration or pure ethanol.^33^ The duration of the evaporation process could be shortened by elongating the droplet contact line by making use of pillars and binary mixtures.^33^ In the same line of research, Wang *et al*. found out that pure liquid droplets evaporate following the two main evaporation modes (CCR and CCA modes) while the binary mixture additionally displays a mixed mode at the end of the evaporation lifetime on a PMMA surface.^21^

Despite the wealth of research on this topic, binary mixture wetting and evaporation on smooth and/or on structured surfaces have been investigated independently for either a smooth/fixed structural parameter with varying surface tension, for a single fluid (mostly pure water), or for a limited range of binary mixture concentrations also on a limited range of solid fraction structures. In this work, we provide a thorough and systematic study addressing a wide range of surface tensions, including pure fluids and their binary mixtures on a wide range of microstructured surfaces with an equal microstructure aspect ratio (fixed pillar diameter and height) varying the spacing between pillars, *i.e.*, the solid fraction. The findings envisaged here provide needful guidelines to predict the evaporative behavior, duration of the different modes as well as the characteristics of the stick-slip mode in terms of contact line jumps, changes in contact angles and pinning forces for fluids varying in surface tension and also the function of the structure of the surface.

## Experimental Method

### Liquid Preparation

**Figure 1:**
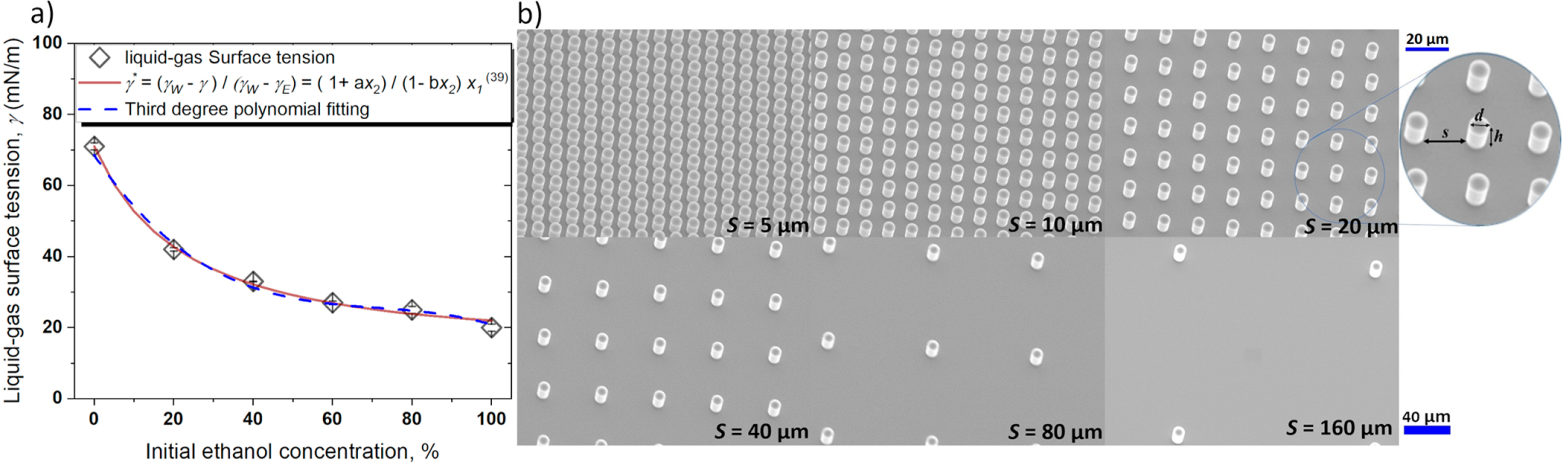
(a) Liquid–gas surface tension, *γ* (mN/m), function of the ethanol concentration by volume (%) for the pure water (0%), pure ethanol (100%), and their binary mixtures as empty rhomboid symbols. The red solid line represents the fitting of eq 2 from Vazquez *et al*.^39^ for *a* = 0.05 and *b* = 0.8, while the blue dashed line represents the polynomial fitting equals −1.02 × 10^–4^*x*^3^ – 0.022*x*^2^ – 1.6*x* + 68.6 = 0. A regression coefficient between our experimental data and eq 2 from Vazquez *et al*.^39^ is 0.994, while that between our experimental data and the polynomial fitting is 0.992. (b) Scanning electron microscopy (SEM) of the surfaces at a 30° tilting angle and at the same magnification (500×), for *h* = 10 μm and *d* = 10 μm micropillars with *s* = 5 μm spacing (top left), *s* = 10 μm spacing (top middle), *s* = 20 μm spacing (top right), *s* = 40 μm spacing (down left), *s* = 80 μm spacing (down middle), and *s* = 160 μm spacing (down right). The scale bar is 40 μm. Inset magnification for *s* = 20 μm with a scale bar of 20 μm. Note that the error bars in panel (a) are of the same size or smaller than those of the symbols represented.

Pure liquids, namely, deionized water (W) and pure ethanol (E) (Sigma-Aldrich), and also their binary mixtures were used in this study. The binary mixtures were prepared on a volume basis as follows: 80% W-20% E, 60% W-40% E, 40% W-60% E, and 20% W-80% E. This allows the effect of the concentration and/or fluid surface tension to be studied. For brevity, the term concentration is used as a proxy for volume percentage of ethanol in some graphs and descriptions below. The surface tension of the different fluids used was measured using the pendant drop method in air in a drop shape analyzer device DSA30 (KRÜSS Gmbh, Hamburgh, Germany) at ambient temperature *T*_amb_ = 22 ± 3° with an ambient relative humidity of 35 ± 8% and at ambient pressure. Care was taken to ensure that preferential evaporation of the most volatile components was minimized. Figure 1a represents the measured liquid–gas surface tensions of the fluids used in this study.^27^ To note here is the gradual increase in the surface tension from 100% ethanol to 20% ethanol, while a sharp increase where the surface tension almost doubles ensues between 20% and pure water. By referring to this trend, we can better understand and explain the effect of surface tension during the evaporation of binary mixture droplets, which will be discussed in the following sections.

### Surface Fabrication and Characterization

A total of six different microstructured surfaces were fabricated and used in this study. Microstructured surfaces have similar cylindrical pillars with 10 μm in height, *h*, and 10 μm in diameter, *d*, for an aspect ratio *h*/*d* = 1. Meanwhile, the spacing between pillars *s* varies as *s* = 5, 10, 20, 40, 80, and 160 μm and the corresponding solid fractions equal 0.34, 0.20, 0.09, 0.031, 0.009, 0.002, respectively. Microstructured surfaces were fabricated on a silicon wafer, purchased from Si-Mat (Silicon Materials, Landsberg, Germany), via deep reactive ion etching (DRIE), and further coated with a hydrophobic self-assembled monolayer at the Scottish Microelectronic Centre (SMC) at the University of Edinburgh (see Al Balushi *et al*.^27^ and the Supporting Information of Zhao *et al*.^38^ for more details on the fabrication and patterning of the resist and DRIE fabrication procedure).

Figure 1b shows high magnification snapshots of the surfaces used in this study for *s* = 5 μm (top left), *s* = 10 μm (top middle), *s* = 20 μm (top right), *s* = 40 μm (down left), *s* = 80 μm (down middle), and *s* = 160 μm (down right) captured by scanning electron microscopy (SEM), in a JSM-IT100 InTouchScope scanning electron microscope from JEOL Ltd., (Japan) with an accelerating voltage of 20 kV, probe current (PC) of 50 a.u., and tilting angle of 30°. The inset in Figure 1b includes magnification of the micro-pillared structures for *s* = 20 μm.

### Wettability and Evaporation Characterization

Droplets with defined volumes of 3 ± 0.2 μL for the different liquids described above were gently deposited at the center of each microstructured surface using a drop shape analyzer device DSA 100 (KRÜSS Gmbh, Hamburgh, Germany). Needles with inner and outer diameters of 0.23 and 0.41 mm, respectively, were purchased from Octoinkjet Ltd. (UK) and attached to a 2 mL syringe placed in the DSA100 automatic dosing system utilized to produce the droplets. All experiments were performed at ambient temperature *T*_amb_ = 22 ± 3° with an ambient relative humidity of 35 ± 8% and at ambient pressure. The apparent contact angles, *θ* (°), on the differently structured surfaces and for the different pure and binary mixture fluids studied in this work are presented in Table 1. The excellent agreement between the contact angles reported here and those reported in refs (27) and (38) is highlighted. The apparent contact angles reported in Table 1 were averaged from at least five independent measurements from different azimuthal directions similarly as reported by Al Balushi *et al*.^27^ The different contact angles reported in Table 1 agreed quantitatively with the classic Cassie–Baxter and partial non-wetting Wenzel equations as reported by Al Balushi *et al*.^27^ We note here that in the presence of a high ethanol concentration equal or above 60%, *i.e.*, fluid surface tension equal or below 30 mN/m and short pillar spacing equal or below *s ≤* 20 μm, droplets display a shape other than symmetric as reported in the work of Al Balushi *et al*.^27^ Higher standard deviations were reported for high ethanol concentrations (60% or above) droplets on surfaces with short spacing (below 40 μm) where asymmetrical droplets were observed. The high standard deviations reported support the different contact angles of asymmetric droplets.

**Table 1:** Apparent Contact Angles, *θ* (°), of Pure Water, Pure Ethanol, and Their Binary Mixtures on the Different Microstructured Surfaces with Different Spacings Utilized in This Study^27^

	ethanol volume percentage (indicative of concentration)					
spacing (*s*)	0%	20%	40%	60%	80%	100%
5 (μm)	147° ± 1	142° ± 1	143° ± 1	122° ± 5	109° ± 4	86° ± 5
10 (μm)	147° ± 1	142° ± 1	143° ± 1	112° ± 4	95° ± 3	51° ± 4
20 (μm)	148° ± 1	146° ± 1	144° ± 1	91° ± 3	78° ± 6	46° ± 4
40 (μm)	112° ± 1	98° ± 2	73° ± 2	70° ± 3	63° ± 1	44° ± 2
80 (μm)	110° ± 1	90° ± 2	64° ± 3	56° ± 5	58° ± 2	45° ± 1
160 (μm)	112° ± 1	73° ± 3	60° ± 2	55° ± 3	59° ± 1	41° ± 2
smooth	111° ± 2	83° ± 2	72° ± 1	67° ± 1	58° ± 1	53° ± 1

After being deposited on the substrate, droplets were left to evaporate fully, and the complete evaporation process was recorded from the side and top views. The contact angle *θ* (°), the volume *V* (μL), the height *h* (mm), and the base radius *R* (mm) of the droplets were then extracted in time *t* (mins) using the drop shape analyzer DSA100 and the DSA1 v1.9 software from KRÜSS (KRÜSS Gmbh, Hamburgh, Germany). Evaporation behavior reported in terms of contact angle *θ* (°) and base radius *R* (mm) in time *t* (mins) includes the average and standard deviation of at least three independent experiments. While the apparent contact angle measurements were carried out from differen azimuthal directions, in the present work, since we focus on the different evaporation regimes ensuing rather than on the asymmetry of the droplets displayed, observations of droplet evaporation were carried from one single azimuthal direction. To note is that in the work of Feng *et al*., looking at asymetric droplet evaproation, no major qualitative differences in the evaporation modes and/or quantiative differences on the duration of the different evaporation modes were reported, which supports adopting one single visualization angle.^36^

## Results and Discussion

### Droplet Evaporation Results

**Figure 2:**
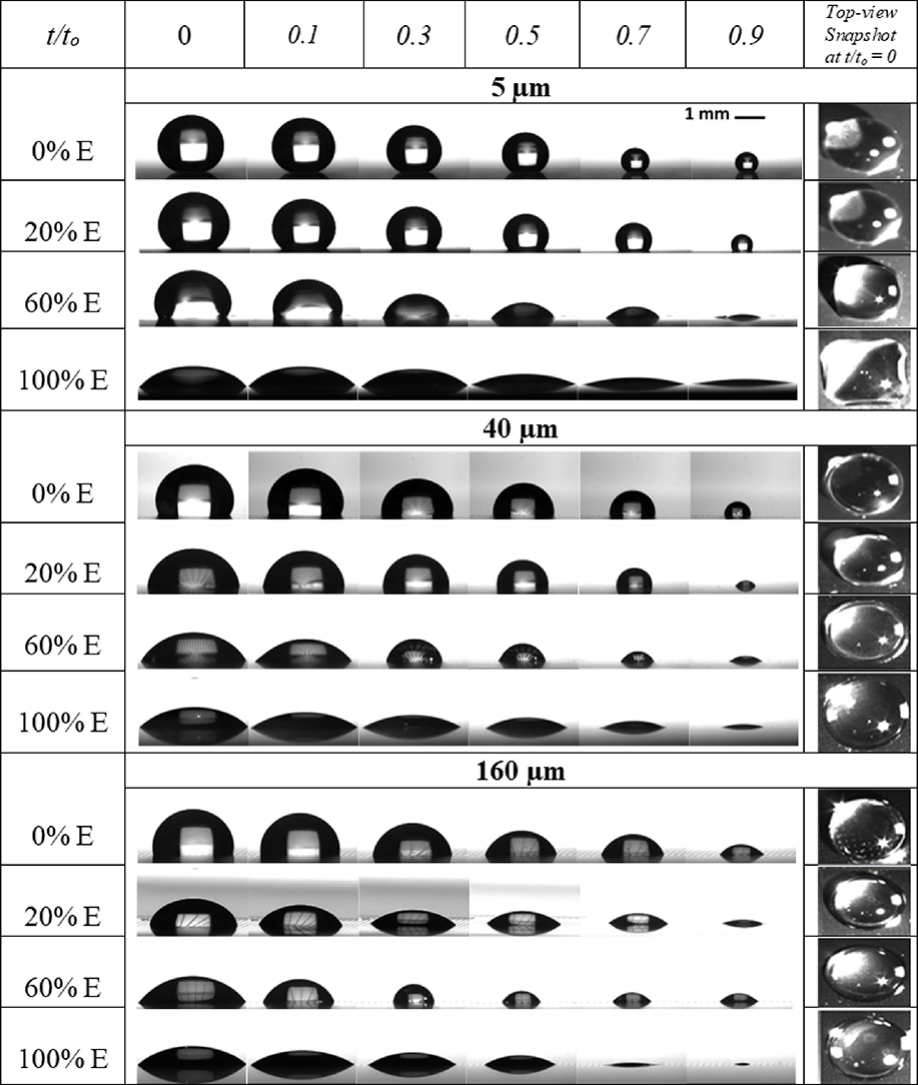
Side view snapshots of droplet profiles at different percentages of the evaporation lifetime (*t*/*t*_0_ = 0, 0.1, 0.3, 0.5, 0.7, and 0.9) on surfaces with different spacings *s* = 5 μm (top), *s* = 40 μm (middle), and *s* = 160 μm (down) for the different binary mixtures (100% W, 80% W-20% E, 40% W-60% E, and 100% E) along with the corresponding top view snapshots at *t*/*t*_0_ = 0. Reproduced or adapted with permission from Al Balushi *et al*. (2022). Copyright 2022 Elsevier.

First, we report experimental observations of the droplet evaporation profile. Figure 2 shows side view snapshots of droplet profiles at different normalized droplet lifetimes with *t* and *t*_0_ as the droplet evaporation time and the total droplet evaporation lifetime, respectively (*t*/*t*_0_ = 0, 0.1, 0.3, 0.5, 0.7, and 0.9), on various pillar spacings *s* = 5, 40, and 160 μm for the following pure and binary mixtures 100% W, 80% W-20% E, 40% W-60% E, and 100% E. In addition, top view snapshots of the droplets at *t*/*t*_0_ = 0 are included to represent the initial droplet shape for each case. To note is that only binary mixtures with ethanol concentrations equal or above 60% on structured surfaces with a spacing below 40 μm were found to display asymmetrical shapes. From Figure 2, the different qualitative evaporation behavior functions of the droplet composition and pillar spacing are evident. Pure water or high water concentration droplets on short pillar spacing display very round spherical droplets with high contact angles during most of the droplet evaporation lifetime. Meanwhile, as the pillar spacing increases, the effect of the pillar spacing is less pronounced and droplets rest with contact angles similar to those on a smooth hydrophobic counterpart with similar evaporation behavior. As the binary mixture concentration increases, droplets display a more wetting behavior and the extent of the different evaporation modes varies.

**Figure 3:**
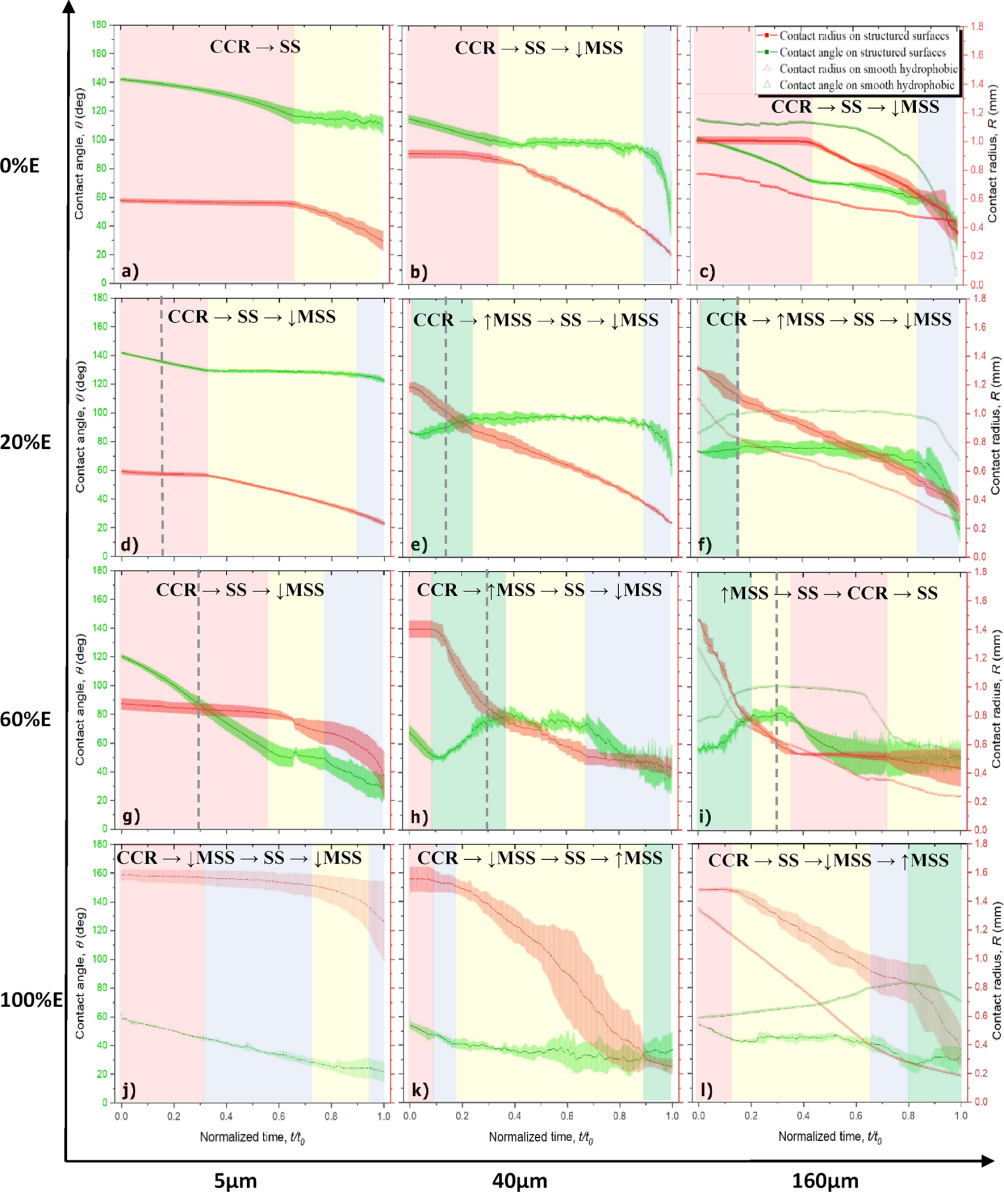
The solid line represents the average evolution of the (dark green) contact angle, *θ* (°), and (dark red) contact radius, *R* (mm), for pure water on (a) 5 μm spacing, (b) 40 μm spacing, and (c) 160 μm spacing, 80% W-20% E binary mixture on (d) 5 μm spacing, (e) 40 μm spacing, and (f) 160 μm spacing, 40% W-60% E binary mixture on (g) 5 μm spacing, (h) 40 μm spacing, and (i) 160 μm spacing, and pure ethanol on (j) 5 μm spacing, (k) 40 μm spacing, and (l) 160 μm spacing, while the shaded area illustrates the standard deviation of at least three independent experiments. Evaporation behavior on the smooth hydrophobic counterpart is included for comparison along with the results for 160 μm spacing. The vertical black dashed line indicates the approximate time at which most of the ethanol has evaporated. Different evaporation modes are defined in colors and by the following abbreviations: pinning mode (red, CCR), stick–slip mode (yellow, SS), increasing contact angle mixed stick–slip mode (green, ↑MSS), and decreasing contact angle mixed stick–slip mode (blue, ↓MSS). Stick–slip modes are identified by analyzing the data for a change in the diameter between 0.005 mm < d*D* < 0.02 mm. The dynamics of droplet evaporation on the other binary mixtures and structured surfaces studied can be found in the Supporting Information.

To provide further qualitative and quantitative analysis on the different evaporation dynamics, Figure 3 shows the evolution of the contact angle, *θ*, and contact radius, *R*, versus normalized time, *t*/*t*_0_, extracted from the same cases represented in Figure 2 and others cases, namely, pure water (0% E), 80% W-20% E, 40% W-60% E, and pure ethanol (100% E) on *s* = 5 μm (left) (shortest spacing in this study), *s* = 40 μm (middle), and *s* = 160 μm (right) (longest spacing in this study) microstructured surfaces. Depending on the structure spacing and the nature of the liquid (in terms of surface tension), different evaporative behaviors ensue, namely, the constant contact radius (CCR), the stick–slip (SS), and the different mixed stick–slip (MSS) modes, as represented in Figure 3, while on the smooth counterpart, the constant contact angle (CCA) mode ensues. The determination of the SS and/or MSS modes was established by looking at the evaporation data for changes in the diameter *D* between 0.005 and 0.02 mm with 0.002 mm as the error on the measurements. The droplet shape and apparent contact angle also vary depending on the structure spacing and the nature of the liquid, with contact angles ranging between 41 and 148°. The dynamics of the contact angle and contact radius are reported as the average value in solid lines and standard deviation, of at least three independent experiments, in shaded areas. The evaporation behavior for each specific mixture evaporating on a smooth hydrophobic substrate is also included for comparison along with the experimental results on *s* = 160 μm. Both the qualitative evaporation mode/s and the quantitative duration of the modes looking from one single azimuthal angle shall be representative of the evaporation even for droplets displaying asymmetric shapes, although the magnitude of the contact angle and contact radius may differ.^36^ Of note is the rather low standard deviation in the case of evaporation on the smooth hydrophobic surface, *i.e.*, the smallest of the shaded areas. The complete droplet evaporative behaviors for the full range of binary mixtures and solid structures utilized in this study can be retrieved within the accompanying Supporting Information. In what follows, the detailed experimental results are presented and discussed.

#### Pure Water

When looking into the wettability and contact angles of pure water on the different structured surfaces studied, a maximum apparent contact angle of 148° is reported on the shortest spacing *s* = 5 μm, whereas on larger spacing surfaces, *i.e.*, 40, 80, and 160 μm, contact angles range between 110 and 112°, which are similar contact angles to those reported on the smooth hydrophobic surface. Droplets of pure water on all surfaces used have a perfect spherical cap and circular footprint with ±1° difference in contact angles when measured from different azimuthal directions.^27^ On the shortest spacing *s* = 5 μm surfaces represented in Figure 3a (and similar to *s* = 10 μm), pure water droplets evaporate following the CCR mode for more than half of the droplet lifetime followed by the stick–slip mode. During this stick–slip mode, the contact angle remains within a rather constant range while the contact radius decreases until the end of the evaporation lifetime, similar to the stick–slip evaporation reported upon the addition of nanoparticles to a fluid.^9^ On larger spacing *s* = 40 and 160 μm represented in Figure 3b and Figure 3c, respectively, three sequential evaporation modes are observed, which are the CCR, stick–slip, and mixed modes. The CCR and stick–slip modes reported for larger spacing are similar to those reported for shorter spacing *s* = 5 μm; however, there are obvious differences in the duration of these evaporation modes, which will be further discussed in subsequent subsections. In addition, when looking more closely into the mixed mode near the end of the droplet lifetime, the contact angle and the contact radius both decrease following a stick–slip fashion, which is different from the mixed mode where both the contact angle and contact radius monotonically decrease in time. We coin this mode as “the decreasing contact angle mixed stick–slip” mode. It is worth noting here that such a mode has not been reported in the literature, presumably due to the lack of resolution and accuracy on the droplet shape analysis at the late stages of droplet evaporation. In the case of pure water on structured surfaces, the larger duration of the CCR mode and the occurrence of stick–slip mode instead of the CCA mode are highlighted as the main differences when comparing to the evaporation of droplets on smooth hydrophobic surfaces represented in Figure 3c. Nonetheless, in the case of large spacing, the strength and frequency of the stick–slip events are minimized as per the reduced number of available pinning sites for the contact line to both pin and depin when compared to shorter spacing.

#### Binary Mixtures

In the case of binary mixtures, the apparent contact angles reported sit between those of pure water and pure ethanol for each of the independent surface structure configurations addressed, as presented in Table 1. When looking into evaporation, as described in the Introduction, the most volatile fluid, in this case, ethanol, evaporates first and then water evaporates.^40^ The reason for the quicker ethanol evaporation is its higher vapor pressure when compared to water under ambient conditions. Three distinctive phases including the transition phase have been proposed as phase I: ethanol evaporates at the beginning of the droplet evaporation; phase II: or transition phase, where the strength of ethanol evaporation diminishes in favor of that of water evaporation; and phase III: where there is mainly pure water left to evaporate. When looking into the droplet profile for ethanol–water binary mixtures, as the most volatile, *i.e.*, lowest surface tension fluid, ethanol evaporates, there is a local increase in surface tension as the concentration of water increases and so does the contact angle of the evaporating droplets. Presumably, most of the ethanol will have evaporated at the point at which the contact angle reaches the highest of the contact angles observed during evaporation.^15^ Hence, to estimate and delimit the different phases, we studied the evaporation results of the different binary mixtures on the smooth hydrophobic surface, and we established a transition threshold between phase I and phase III at the point at which the contact angle reaches the highest of the contact angle reported, which is represented with a vertical dashed black line in Figure 3, which is quantified as 17 and 30% of the droplet lifetime for 20 and 60% ethanol–water concentrations, respectively. Moreover, when looking at the evaporation rates of the evaporating binary mixture droplets and assuming that only ethanol evaporates, the mentioned thresholds can be quantified as 18.7 ± 1.2 and 29 ± 2.9%, which is in rather good agreement with those represented in Figure 3. Moreover, to support that most of the ethanol evaporates first, Pereira *et al*. made use of smart sensors to record the concentration of ethanol during the evaporation of ethanol–water binary mixtures on hydrophilic surfaces.^41^ They addressed the evaporation of ethanol–water binary mixtures with concentrations ranging between 0 and 100%. They observed that for 70% ethanol–water binary mixtures, the ethanol concentration drops to nearly 5% or less within 15–40% of the droplet lifetime.

#### Binary Mixtures 20% Ethanol

For the case of 80% W-20% E, droplets show symmetric geometry on all surfaces with a perfect circular footprint and a spherical cap.^27^ On 5 μm spacing (Figure 3d), the apparent contact angle is approximately 142°, evaporation then begins in the CCR mode followed by a stick–slip mode for almost 90% of the droplet lifetime, and then the droplet shifts to the mixed stick–slip mode for the rest of the evaporation lifetime. During ethanol evaporation, *i.e.*, the initial 15% of the droplet lifetime, the droplet evaporates solely in the CCR mode. Once most of the ethanol has evaporated, the droplet continues evaporating in the CCR mode until 30% of the droplet’s lifetime. Thereafter, the contact line recedes while the contact angle oscillates within a certain range in a stick–slip fashion for 60% of the droplet’s lifetime. Finally, both the contact angle and contact radius recede following the decreasing contact angle mixed stick–slip mode for the remaining 10% of the droplet lifetime. Evaporation behavior for 20% ethanol resembles that of pure water on 5 μm except for the duration of the evaporating modes, which will be discussed in the next section.

However, on larger spacing micropillared surfaces *s* = 40 μm represented in Figure 3e, the evaporation behavior differs from that on shorter pillars *s* < 40 μm. On these surfaces, evaporation initiates in the CCR mode for a rather short time and then transitions into a different than the classical stick–slip evaporation mode observed for pure fluids where the contact angle oscillates within a constant range or from the decreasing contact angle mixed stick–slip mode where both contact angle and contact radius decrease as introduced above. Here, a decrease in the contact radius and a simultaneous increase in the contact angle are observed, which we call the increasing contact angle mixed stick–slip mode. Hence, in this work, we differentiate two different mixed stick–slip modes. On one hand, the decreasing contact angle mixed stick–slip mode occurs at the end of the evaporation lifetime when both the contact angle and contact radius decrease with time in a stick–slip fashion, which is highlighted with blue shading, and it is attributed to pure water evaporation on the structured surfaces. On the other hand, the increasing contact angle mixed stick–slip mode ensues during ethanol evaporation from a binary mixture. During the increasing contact angle mixed stick–slip mode, the contact angle increases while the contact radius decreases following a stick–slip fashion, which is highlighted in green shading and is attributed to the preferential ethanol evaporation and hence to the increase in the droplet surface tension as the droplet becomes richer in high surface tension water. After most of the ethanol evaporates, the droplet continues to evaporate following the increasing contact angle mixed stick–slip mode and shortly after it follows the traditional stick–slip behavior (contact angle oscillates within the same range) and finishes with the decreasing contact angle mixed stick–slip mode.

When looking into a larger spacing of 160 μm in Figure 3f, droplets evaporate initially in the CCR mode for approximately 10% of the droplet lifetime and then following the traditional stick–slip mode where the contact radius decreases while the contact angle remains within a certain constant range for more than 80% of the droplet lifetime. Initially, and until ethanol completely evaporates, a slight increase in the contact angle in an increasing contact angle mixed stick–slip mode is inferred. At the end of the evaporation, as per pure water cases, droplets evaporate following the decreasing contact angle mixed stick–slip mode. Comparing the same binary mixture droplet on a smooth hydrophobic surface, a similar general trend where the droplet evaporates with a decrease in the base radius while the contact angle increases for a certain time until, presumably, all ethanol has evaporated is reported. Thereafter, the droplet evaporates following the CCA mode and ends with the mixed mode. The resemblance in the duration of the different evaporation modes as well as on the evaporation modes themselves for pure water and low concentration binary mixture on large spacing surfaces compared to smooth surfaces, except for the presence of stick–slip, is remarkable. The presence of the pillared structures induces pinning, and hence, the stick–slip behavior observed also allows for the increasing and decreasing contact angle mixed stick–slip modes reported.

#### Binary Mixtures 60% Ethanol

For the binary mixture, 40% W-60% E on short spacing surfaces of 5 μm, droplets display an octagonal/squared polygonal shape with an apparent contact angle of around 122° and a standard deviation of 5° when considering the different azimuthal directions. The evaporation behavior for this case is represented in Figure 3g, where a steep decrease in the contact angle for just more than half of the droplet lifetime is observed, while the base radius remains constant despite the occasional minor occurrence of stick–slip behavior. Thereafter, the traditional stick–slip mode is followed by the decreasing contact angle mixed stick–slip mode at the end of the evaporation. Of note is that under these conditions, the decreasing contact angle mixed stick–slip mode ensues, which was not observed in the case of pure water, with a longer duration than for the 20% ethanol mixture on the same micropillared structure. The different subsequent evaporation modes and occurrence of the decreasing contact angle mixed stick–slip mode differ from the traditional stick–slip mode reported for pure water, presumably due to the different initial wetting regimes as partial non-wetting Wenzel and Cassie–Baxter, respectively, the former exerting greater pinning of the contact line. In addition, the initial evaporation in the CCR mode is similar in terms of duration to that of pure water and 80% W-20% E on 5 μm spacing while the magnitude of the contact angle change Δ*θ* is considerably different, which will be further considered and discussed in the next subsections.

When considering the intermediate 40 μm spacing represented in Figure 3h, droplets display a spherical cap shape with evaporation initiating in the CCR mode for approximately 10% of the droplet evaporation lifetime. Thereafter, the droplet contact angle increases in a stick–slip fashion coupled with the decrease in radius, *i.e.*, increasing contact angle mixed stick–slip mode, followed by the traditional stick–slip mode; and the droplet eventually vanishes following the decreasing contact angle mixed stick–slip mode. The duration of each of the different evaporative modes is approximately 30% of the droplet lifetime for each of these modes. Considering now the largest of the spacing of 160 μm represented in Figure 3i, the evaporative behavior of these spherical cap shape droplets differs from that on intermediate and short micropillar spacing where the absence of the CCR mode at the beginning of the evaporation is highlighted. Evaporation of spherical cap droplets begins with the increasing contact angle mixed stick–slip mode as in the case of 20% ethanol, though the duration of this mode is longer for 60 and 80% ethanol–water mixtures as a consequence of the larger amount of evaporating ethanol. This mode is thereafter followed by the traditional stick–slip mode, and once most of the ethanol has evaporated, then the CCR mode with the occurrence of a very small stick–slip phenomenon ensues followed by the traditional stick–slip mode for the last 30% of the evaporation. It is highlighted that when most of the ethanol has evaporated, after 30% of the droplet evaporation lifetime, the droplet tends to adopt a similar qualitative and quantitative behavior to that on the smooth surface, though the CCR mode is absent in this latter.

#### Pure Ethanol

For pure ethanol droplets, the apparent contact angles vary between 86 and 41° as the spacings vary between *s* = 5 and 160 μm, respectively. For pure ethanol on short spacing structures *s* = 5 μm, despite the polygonal shape displayed, the evaporation behavior resembles, to some extent, that of water and the other binary mixtures represented in Figure 3a,d,g where the droplet evaporates in the CCR mode for 30% of the droplet lifetime. Thereafter, the CCR mode shifts into the stick–slip mode, and finally, it vanishes with a decreasing contact angle mixed stick–slip mode. Nonetheless, instead of the traditional stick–slip mode, the decreasing contact angle mixed stick–slip mode ensues under this configuration right after the CCR mode for 40% of the droplet lifetime and then into the traditional stick–slip mode. On the intermediate spacing *s* = 40 μm represented in Figure 3k, spherical cap droplets initially evaporate in the CCR mode for 10% of the droplet lifetime followed by the decreasing contact angle mixed stick–slip mode for the next 10% of the droplet lifetime. Thereafter, it adopts a traditional stick–slip mode for 70% of the droplet lifetime with a final stage where the increasing contact angle mixed stick–slip mode concludes the evaporation. The increasing contact angle mixed stick–slip mode reported here is attributed to the presence of water within the droplet as a consequence of the adsorption-absorption and/or condensation taking place during ethanol evaporation in the presence of relative humidity.^14,15^

On large spacing *s* = 160 μm represented in Figure 3l, the evaporation of these spherical cap droplets initially takes place in the CCR mode, *i.e.*, 12% of the droplet lifetime, and then transitions into the traditional stick–slip mode. At the end of the evaporation, first, a decreasing contact angle mixed stick–slip mode ensues followed by an increasing contact angle mixed stick–slip mode also due to the presence of adsorbed-absorbed and/or condensed water onto thde droplet during pure ethanol evaporation. Pure ethanol droplets on a smooth hydrophobic surface show similar behavior to those on a structured surface when *s* = 160 μm, especially when looking at the decrease in the radius throughout most of the evaporation lifetime. Of note is the absence of the CCR mode on the smooth surface, the increase in contact angle, and the decrease in radius taking place on the smooth surface as earlier reported here and in the literature owed to the adsorption-absorption and/or condensation of water onto the pure ethanol droplet^14,15^ when compared to the traditional stick–slip ensuing on the structured surface, this latter being attributed to the presence of pillars as mentioned before.

### Discussion

Next, we quantify and discuss the different evaporation modes and mechanisms taking place, which were presented and introduced in the previous subsection. In addition, we provide some general guidelines on the initial and main evaporation modes underpinning the phase change of the wide range of fluid surface tensions on a wide range of structured surfaces. To understand how the structured surfaces and fluid binary mixture affect the evaporation dynamics, we first evaluate the duration of each evaporation mode as normalized time introduced in the previous section. Second, we additionally assess and compare the magnitudes of the droplet contact line jumps or slips, δ*D*, and contact angle changes, δ*θ*, as well as evaluation of the pinning force, δ*F*, for each of the configuration studied over the entire evaporation process.

### Duration of Evaporation Modes

**Figure 4:**
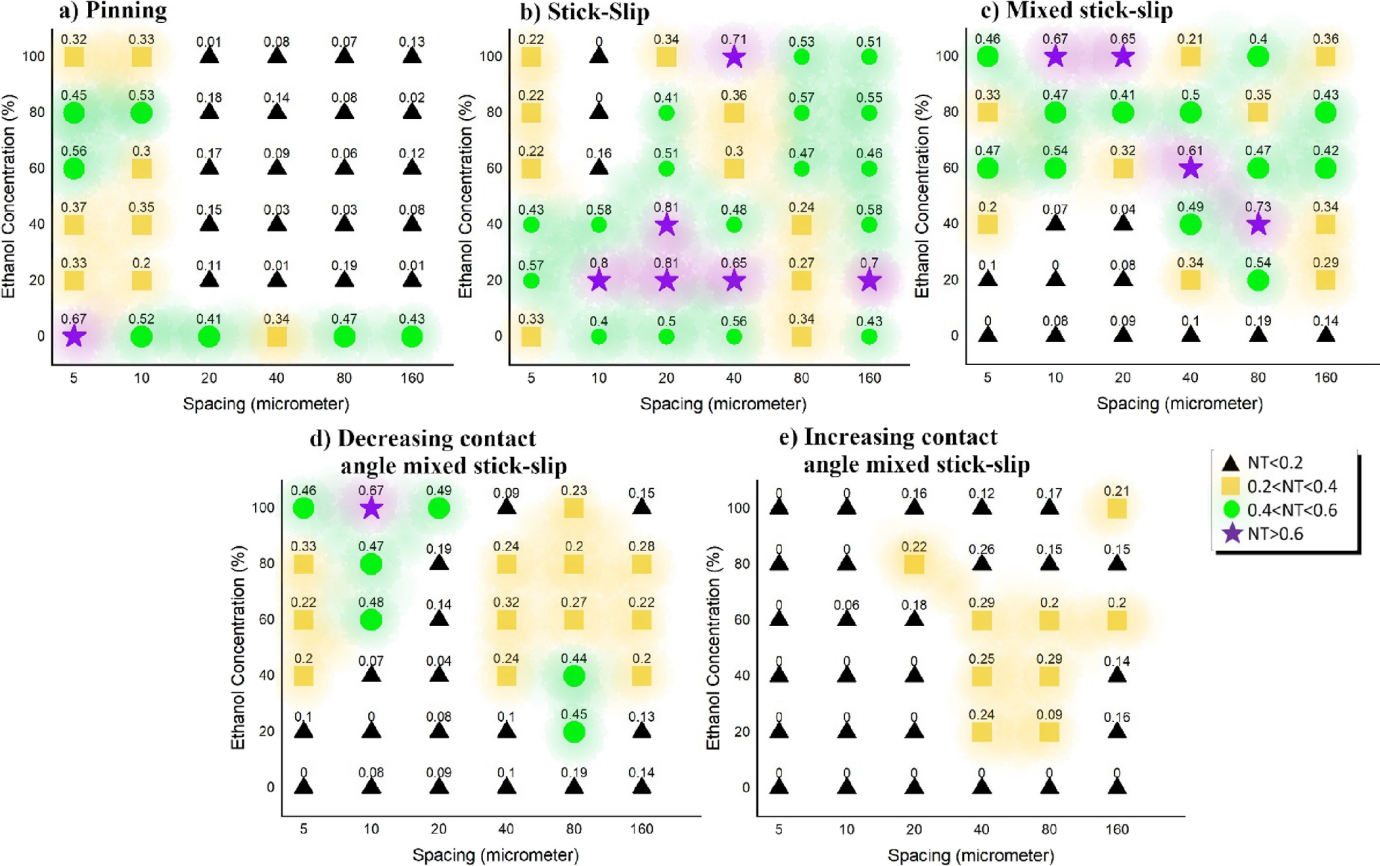
Percentage duration (%) of normalized time (NT) of the (a) constant contact radius (CCR) or pinning mode, (b) traditional stick–slip mode, and (c) newly here reported mixed stick–slip modes. Within the total normalized time for the mixed stick–slip mode, we differentiate between (d) decreasing contact angle mixed stick–slip mode and (e) increasing contact angle mixed stick–slip mode for 0, 20, 40, 60, 80, and 100% E droplets on 5, 10, 20, 40, 80, and 160 μm. The percentage duration (%) of the normalized time is classified by the color and shape map: NT < 0.2 (black triangles), 0.2 ≤ NT < 0.4 (yellow square), 0.4 ≤ NT < 0.6 (green circles), and NT ≥ 0.6 (violet stars). Note that normalized times reported in (c) mixed stick–slip mode are the addition of both normalized times reported in (d) decreasing contact angle mixed stick–slip mode and (e) increasing contact angle mixed stick–slip mode.

Looking at the experimental contact angle and base diameter or radius, the duration of each of the different evaporation modes as normalized times (NT) is calculated for at least three independent evaporating droplets under the same conditions. The percentage duration of the three main distinctive evaporative behaviors reported in this work, namely, CCR mode (pinning), stick–slip mode, and mixed stick–slip modes (this latter mode including both increasing contact angle mixed stick–slip mode and decreasing contact angle mixed stick–slip mode), for each fluid composition and for the different spacings studied, is presented in Figure 4. Note that although some earlier works in the literature report on the occurrence of CCA^25,32,35,42^ mode during evaporation on structured surfaces; nonethless, structures induce a certain degree of pinning, *i.e.*, CCR, stick–slip, or mixed stick–slip modes, and as such, the CCA mode does not ensue in the present case and as such is not reported within Figure 4. Earlier reports on the CCA on such surfaces are mainly due to the lack of spatial and temporal resolution on the droplet profile observations in time. The different thresholds of normalized times in Figure 4 have been chosen to be able to identify the controlling regime, *i.e.*, durations above 60% of the droplet lifetime as well as the presence of other less prevalent regimes of shorter duration from 0 to 20, 20 to 40, and 40 to 60%, ensuing among the CCR, stick–slip and mixed modes, respectively.

#### Pinning CCR Mode

Independently of the droplet composition and spacing between pillars, most/all of the evaporating behaviors begin in the CCR (pinning) mode, as reported in Figure 4a. When looking into the duration of this mode, higher pinning durations occur for all fluids, *i.e.*, pure water, pure ethanol, and their mixtures, on short pillar spacing, *i.e.*, *s =* 5 and 10 μm, while the greatest pinning durations are found for pure water. The CCR mode duration ranges between 20 and 70% of the total droplet evaporation lifetime for these cases. However, pure water shows higher pinning times on all spacings, particularly on *s =* 5 μm, where the longest pinning time occurs for around 70% of the droplet lifetime. As the spacing between structures increases or the density of pillars decreases, *i.e.*, *s* ≥ 20 μm, pinning times below 20% of the droplet lifetime are reported for pure ethanol and the binary mixtures studied while longer pinning times between 20 and 50% are still ensuing in the case of pure water. Thereafter, the evaporation behavior transitions into the stick–slip mode or one of the mixed stick–slip modes.

#### Stick–Slip Mode

When examining the stick–slip mode in Figure 4b, an opposite trend to that reported in Figure 4a is observed, where longer stick–slip times are reported for those configurations where the CCR mode is shorter. The highest duration of the stick–slip mode is seen in the middle of the chart, *i.e.*, for binary mixtures on intermediate spacing. Looking closely at each case, for low ethanol concentration binary mixtures ≤40% E, the stick–slip mode ranges between 40 and 60% of the droplet lifetime on all surfaces with different spacings. Meanwhile, for high ethanol concentrations ≥60% E, the duration of the stick–slip mode is the longest on surfaces with *s* ≥ 20 μm, while for short spacing *s* < 20 μm, the stick–slip mode duration was the shortest. For the cases of pure water droplets, unlike pure ethanol, stick–slip behavior takes place between 33 and 56% of the evaporation lifetime independently of the spacing. For pure ethanol, the stick–slip behavior occurred on the surfaces with large spacing *s* ≥ 40 μm for the duration between 51 and 71% of the droplet lifetime.

#### Mixed Stick–Slip Modes

The rest of the evaporation, in fact, ensues in a stick–slip mode behavior coupled with the decrease or increase in the contact angle, or as defined earlier in the mixed stick–slip mode, which has been represented in Figure 4c and is independent of the stick–slip results presented in Figure 4b. This distinctive evaporation mode has been observed on most of the configurations mainly at the end of the evaporation while it is more obvious for high ethanol concentration binary mixtures ≥60% E and for pure ethanol, independently of the micropillar spacing, as represented in Figure 4c. Meanwhile, low concentration binary mixtures ≤40% E show mixed stick–slip behavior only on surfaces with large spacing *s* ≥ 40 μm. In the case of pure water, the duration of the mixed stick–slip mode with respect to the droplet lifetime was the shortest. The short duration of the mixed stick–slip mode also ensues for low ethanol concentration droplets ≤40% E on surfaces with short spacing *s* ≤ 20 μm, which lasts for less than 20% of the evaporation lifetime and/or does not ensue on particular cases.

On one hand, looking closely at the mixed stick–slip evaporation mode, Figure 4d and Figure 4e additionally allows for distinguishing between the decreasing contact angle mixed stick–slip mode and the increasing contact angle mixed stick–slip mode, respectively. Regarding the decreasing contact angle mixed stick–slip mode, it is found that the duration of this mode with respect to the droplet lifetime is longer for the binary mixtures and pure ethanol than for pure water cases independently of the different structured surfaces used. This mode typically ensues at the end of the droplet lifetime for most/all cases as a consequence of the presence of the structures that pin the droplet contact line further, and as such, both the contact angle and the contact radius decrease to account for evaporation. For all pure water and binary mixture cases, the following commonality applies: when the contact radius reaches a value equal or below 0.6 mm on surfaces with *s* = 40 and 160 μm spacing, as represented in Figure 3b,c,e,f,h,i, the decreasing contact angle mixed stick–slip mode takes place. This same behavior occurred for the same binary mixtures on all surfaces with spacing *s* ≥ 20 μm as it can be retrieved from the Supporting Information. For pure ethanol, this mode was observed both at the middle and at the end of the evaporation lifetime without clear indication or relation to the droplet size.

On the other hand, the increasing contact angle mixed stick–slip mode mainly occurs for binary mixture droplets on large spacing, although with a duration shorter than 30% of the droplet lifetime. The increasing contact angle mixed stick–slip mode ensues just after the CCR mode at the beginning of the droplet evaporation, and this is due to the evaporation of ethanol resulting in the droplet surface tension increase^12,13,15,26^ and hence the contact angle increase coupled with stick–slip behavior due to the presence of the structures. For pure ethanol, this mode occurs due to the adsorption-absorption and/or condensation of water vapor onto the surface and hence the transition from pure ethanol to a binary mixture and/or to pure water, which eventually changes locally the droplet surface tension.^15^

### Stick–Slip Contact Line (CL) Jumps and Contact Angle Changes

**Figure 5:**
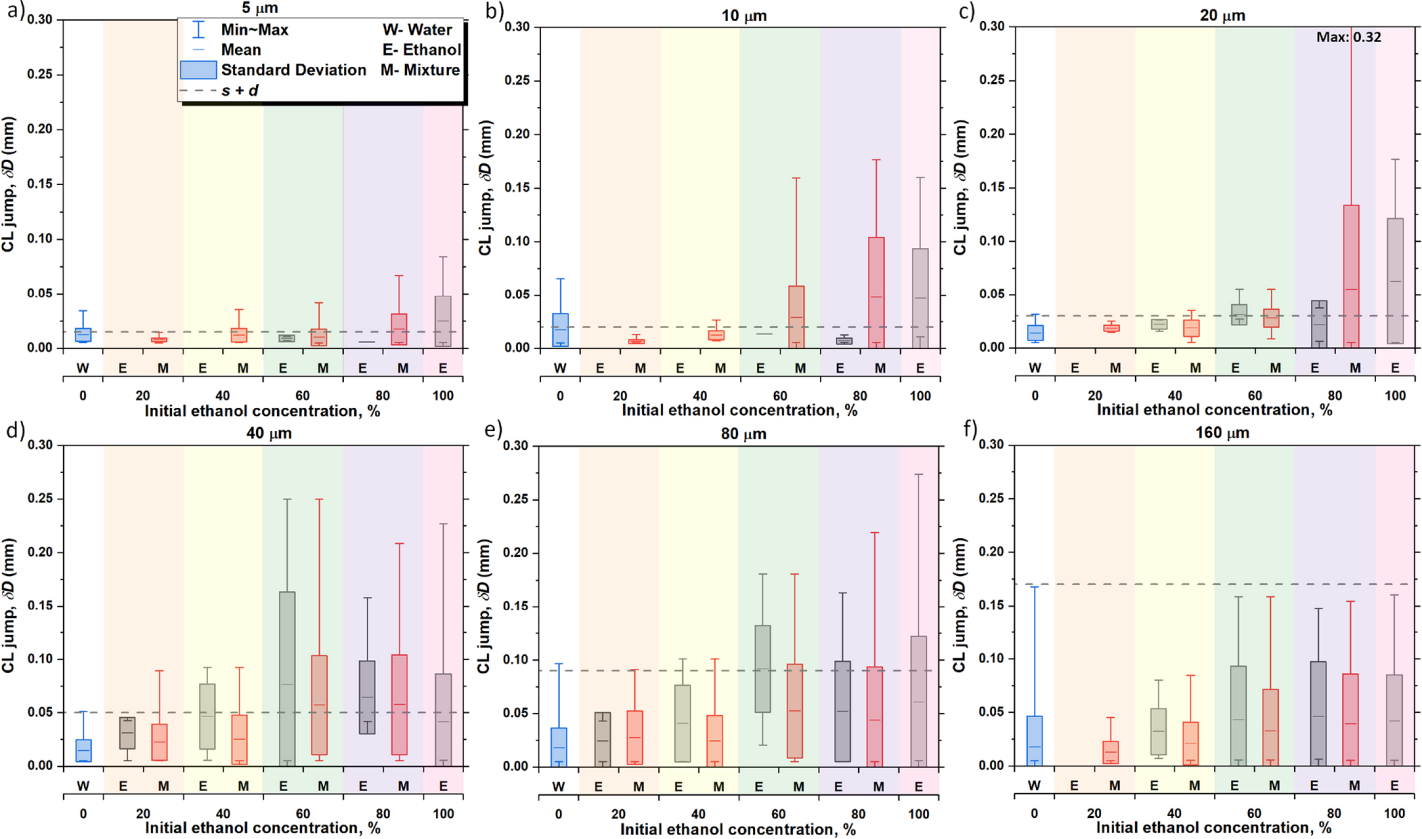
Average jump distance, δ*D* (mm), of the contact line (CL) for the different pure fluids and binary mixture concentrations on (a) 5 μm, (b) 10 μm, (c) 20 μm, (d) 40 μm, (e) 80 μm, and (f) 160 μm pillar spacing. Solid horizontal lines within the closed boxes represent the average, closed box columns represent the standard deviation, and whiskers represent the maximum and minimum values observed. Black columns represent preferential ethanol evaporation (E) and phase I, blue columns represent pure water (W), red columns represent both ethanol and water evaporation from the different binary mixtures (M), while gray dashed lines show the expected jump distance equals *s* + *d*. % indicates the initial ethanol concentration. The different background shaded areas represent the six fluids used in this study. Note that the average jump distance, δ*D* (mm), and standard deviation were calculated from the local jumps taking place for three independent experiments rather than by making use of the averaged droplet profiles reported in Figure 3.

**Figure 6:**
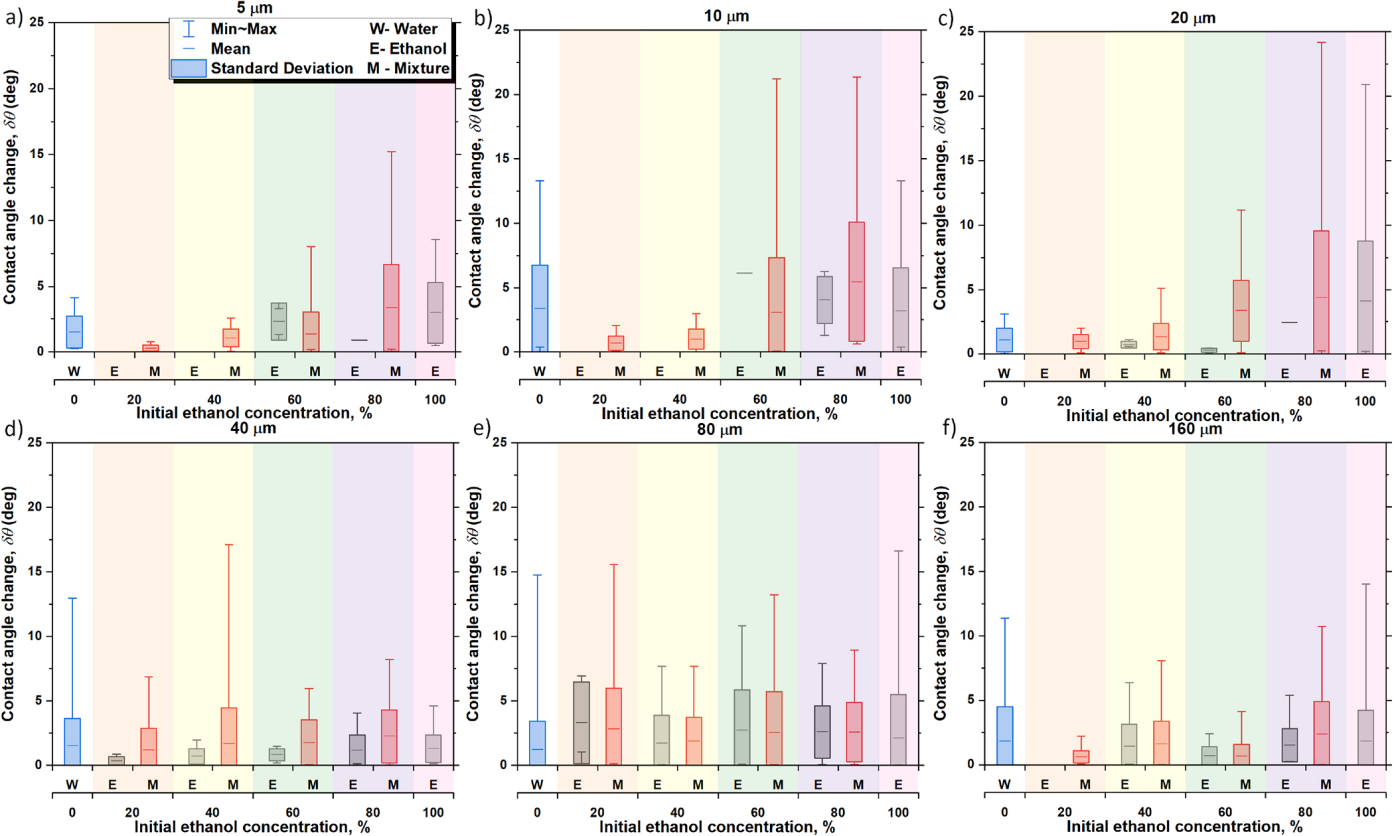
Average change in the contact angle, δ*θ* (°), for the different pure fluids and binary mixture concentrations on (a) 5 μm, (b) 10 μm, (c) 20 μm, (d) 40 μm, (e) 80 μm, and (f) 160 μm. Solid horizontal lines within the closed boxes represent the average, the closed box columns represent the standard deviation, and whiskers represent the maximum and minimum value of the change in the contact angle. Black columns represent preferential ethanol evaporation (E) and phase I, blue columns represent pure water (W), red columns represent both ethanol and water evaporation from the different binary mixtures (M), while gray dashed lines show the expected jump distance equals *s* + *d*. The different background shaded areas represent the six fluids used in this study. Note that the average contact angle, δ*θ* (°), and standard deviation were calculated from the local jumps taking place for three independent experiments rather than by making use of the average droplet profiles reported in Figure 3.

This section focuses on the discussion of the contact line behavior during the different pinning/depinning events taking place in the stick–slip evaporating mode excluding the CCR mode. Figures 5 and 6 compare the average distance of the contact line jumps, δ*D*, and the average changes in contact angle, δ*θ*, respectively, for the different concentrations and different structured surfaces investigated. Since for binary mixtures and for pure ethanol, the droplet concentration changes in time, to provide deeper insights on the different stick–slip evaporating behavior, the magnitude of the jumps and that of changes in contact angle are differentiated whether ethanol preferentially evaporates (E) or for the complete binary mixture evaporation including both water, ethanol and transition evaporation periods or stages (M). Hence, black columns (E) represent phase I assuming sole ethanol evaporation, which typically occurs at the first stage of the binary mixture evaporation due to the greater volatility of ethanol when compared to water,^26,40^ and red columns (M) represent the average values over the complete binary mixture evaporation process without distinguishing the evaporating component, *i.e.*, ethanol and/or water evaporating. We note here that results on the average distance of the contact line jumps, δ*D*, and the average change in contact angle, δ*θ*, assuming that only water evaporates, *i.e.*, phase II, are in good quantitative agreement with those results represented for the mixture (M). Hence, for simplicity, we only represent results for ethanol and for the mixture within subsequent Figures 5 and 6 while the complete results showing ethanol (E), water (W), and the complete binary mixture evaporation (M) can be found in the accompanying Supporting Information, S2: Stick–slip contact line (CL) Jumps and contact angle changes in Figures S7 and S8 .

The spacing plus the pillar diameter, calculated as *s* + *d*, accounts for the expected jump distance to overcome one row of micropillars so that the contact line sits at the subsequent micropillar row, which is mainly function of the pillar spacing *s* and is represented as a horizontal dashed lines in Figure 5. For short spacing (*s* = 5, 10, and 20 μm) substrates shown in Figure 5a–c, the magnitude of the droplet contact line discrete jumps, δ*D*, is highly dependent of the spacing between pillars with jumps of similar values as *s* + *d* for both the pure fluids and the binary mixtures studied. Of note is the remarkable agreement between the magnitude of the jumps for pure water (W) and for the binary mixture (M) with concentrations of 40 and 60% E evaporating and the expected jump distance *s* + *d*.

As the spacing between pillars increases, *i.e.*, *s* ≥ 40 μm, the number/density of microstructures or sites available for contact line pinning decreases, and the contact line is able to move more freely with the consequent mismatch between the expected jump distances and the measured ones, as shown in Figure 5d–f. The magnitudes of the jumps are lower than the expected *s* + *d* for most of the binary mixture concentrations reported for large spacing above *s* ≥ 40 μm either considering preferential ethanol evaporation in phase I (E) or both phases considered together (M).

At the same time, following volume conservation, the depinning of the contact line causes an increase in the contact angle during each stick–slip occurrence. The change in contact angle, δ*θ*, for the different pillar spacing and binary mixture concentrations is presented in Figure 6. As a general trend, the magnitude of the change in contact angle decreases with the ethanol concentration for short spacing *s* ≤ 20 μm during most of the phases reported, *i.e.*, independently of the assumption whether pure ethanol (phase I) preferentially evaporates or taking into consideration the evaporation of both water and ethanol (M). The measured and reported changes in contact angle, δ*θ*, reported in Figure 6 can be then used for the estimation of the pinning forces involved during the evaporation of the different binary mixtures on different solid fraction surfaces, which are presented next.

### Pinning/Depinning Force δ*F*

The magnitude of the contact line jumps and/or changes in contact angle are attributed to the pinning/depinning events as a consequence of the pinning force imposed by the structures at the contact line, which in turn increases the excess surface free energy of the droplet as its shape deviates from spherical cap during evaporation.^9,43^ By establishing a 2D pinning force balance at the contact line based on the change in contact angle δ*θ* during the pinning stage, the pinning/depinning force, δ*F*, displayed in eq 1 provides quantification on the force per unit of length acting at the contact line:^9,43^
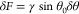
1where *γ* is the liquid–gas surface tension and *θ*_0_ is the initial apparent intrinsic contact angle.

**Figure 7:**
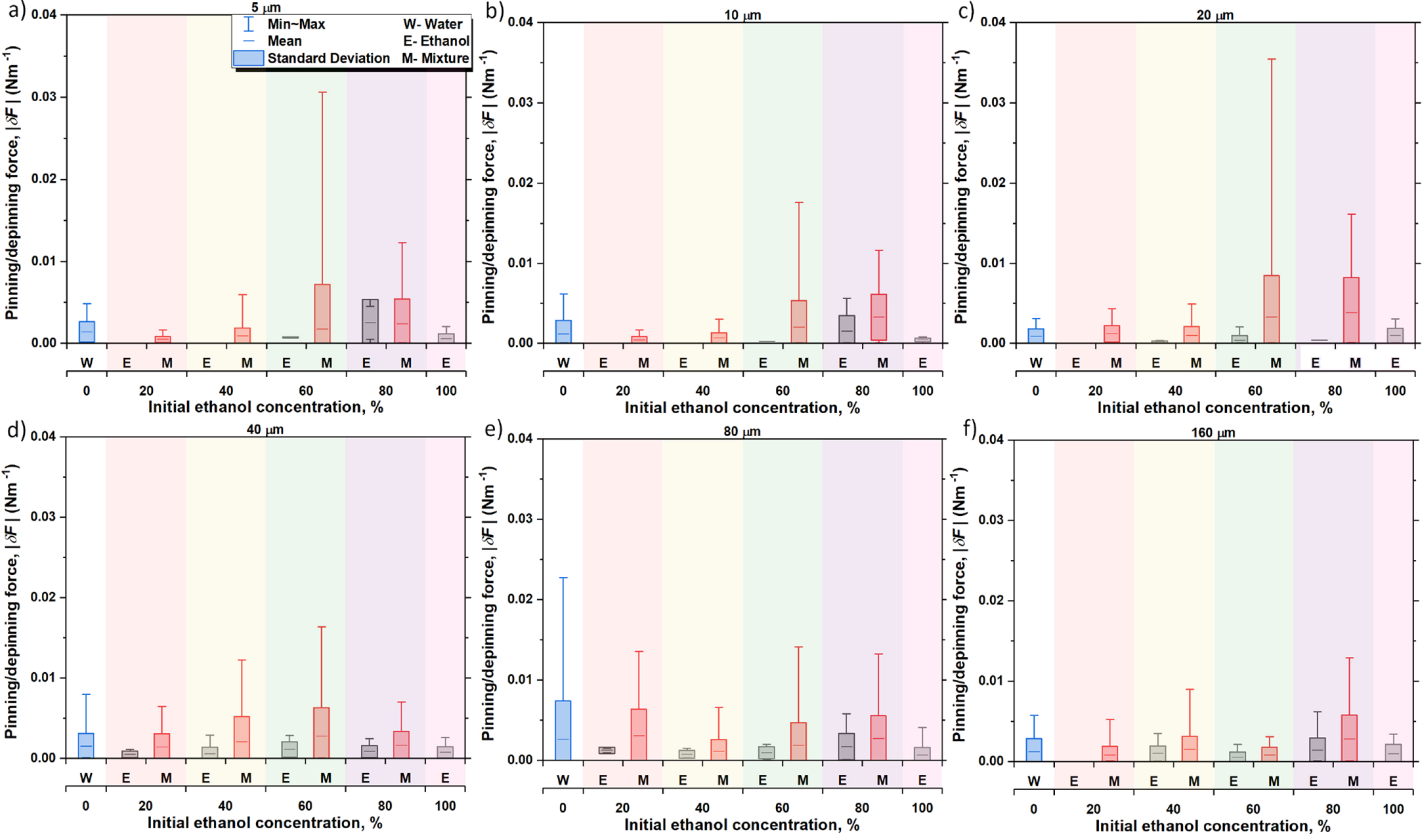
Average pinning/depinning force, δ*F* (N/m), for the different pure fluids and binary mixtures on (a) 5 μm, (b) 10 μm, (c) 20 μm, (d) 40 μm, (e) 80 μm, and (f) 160 μm. Solid horizontal lines within the closed boxes represent the average, the closed box columns represent the standard deviation, and whiskers represent the maximum and minimum value of the change in the pinning/depinning force. Black columns represent preferential ethanol evaporation (E) and phase I, blue columns represent pure water (W), and red columns represent both ethanol and water evaporation from different binary mixtures (M). The different background shaded areas represent the six fluids used in this study. Note that the average pinning/depinning force, δ*F* (N/m), and standard deviation were calculated from the local jumps taking place for three independent experiments rather than by making use of the average droplet profiles reported in Figure 3.

The 2D force balance established in eq 1 provides a more reliable metric on quantifying the different forces acting at the contact line function of the effect of both fluid surface tension and pillar spacing, which are reported in Figure 7 below. Note that calculations on the pinning/depinning force making use of eq 1 focus on the stick–slip evaporating mode excluding the CCR mode, and as such, *θ*_0_ is approximated as the average of the initial contact angles during evaporation in the stick–slip period. This is valid since most of the stick–slip events occur within the same range of contact angles, except for the cases where the droplets evaporate in the increasing or decreasing contact angle mixed stick–slip mode. We also note here that the average pinning/depinning force, δ*F*, assuming that only water evaporates after almost all ethanol has evaporated, *i.e.*, phase III, and those for the total mixture (M) evaporation are in good quantitative agreement. Hence, for simplicity, we only represent results for ethanol and for the mixture within Figure 7 while the complete results showing ethanol (E), water (W), and the complete binary mixture evaporation (M) can be found in the accompanying Supporting Information, S3: Pinning/depinning force δF in Figure S9.

When looking at the average pinning/depinning force, δ*F* , calculated and reported in Figure 7, all values reported for all surface structures and pure fluids or binary mixtures are within 0.005 N/m, which is almost an order of magnitude smaller than pinning/depinning force values reported for pure water on smooth hydrophilic substrates during the first pinning stage or CCR mode reported in the work of Orejon *et al*.^9^ In the present case, pinning/depinning force, δ*F*, is attributed to the presence of structures. Despite the different number and density of microstructures (see Figure 1b) and the different duration of the different evaporating modes, including the stick–slip mode reported in Figure 4, no major differences or trends are found when comparing the different pillar spacing and the pure fluids with most values within 0.002 N/m. Slightly higher values are found in the case of pure water when compared to pure ethanol owing to the greater surface tension of the former.

However, when looking into the binary mixtures, low ethanol concentration fluids ≤40% E show certain dependency on the spacing between pillars. On one hand, on short spacing, *s* ≤ 20 μm surfaces, low ethanol concentration ≤40% E binary mixtures show the smallest magnitude of the pinning/depinning force, δ*F*, compared to the rest of the fluids studied. Meanwhile, as the ethanol concentration increases, the pinning/depinning force, δ*F*, increases with the lowest values for 20% E and the highest values for 80% E. The increasing pinning/depinning force, δ*F*, with ethanol concentration is attributed to the different wetting regimes displayed by the droplets where for pure water and low ethanol concentrations, *i.e.*, ≤40% E, droplets initially display the Cassie–Baxter non-wetting regime,^27^ whereas for high ethanol concentrations, *i.e.,* ≥60% E, droplets initially display the partial non-wetting Wenzel regime^27^ with the consequent higher pinning/depinning forces, δ*F*. Of note is the rather large standard deviations observed for short spacing *s* ≤ 20 and high ethanol concentrations ≥60% E, presumably due to the different geometrical shapes displaying more wetting droplets,^27^ as represented in Figure 2. On the other hand, on large spacing surfaces *s* ≥ 40 μm, average pinning/depinning force, δ*F*, values do not display a clear trend and most values are within the standard deviations reported. For such large spacings *s* ≥ 40 μm, the presence of structures seems to not influence considerably the pinning of the contact line, hence the low pinning force values, which is supported by the similar wetting^27^ and evaporation behavior as those displayed on the smooth hydrophobic surface in Figure 3.

Overall, the rather similar pinning/depinning forces, δ*F*, independently of the surface spacing reported, which are an order of magnitude smaller to those reported on smooth hydrophilic substrates, are plausible to some extent by acknowledging that the characteristic size of the structures is at least 1–2 orders of magnitude smaller than the droplet size. The small influence of the surface structures on the overall pinning/depinning force, δ*F*, becomes more apparent when looking at the large spacing where the droplets typically behave as on the smooth flat counterpart both in terms of contact line pinning and pinning/depinning forces, δ*F*.

Meanwhile, the different type of fluid does impact the magnitude of the pinning/depinning forces, δF, as per the different wetting regimes and evaporation behaviors reported. The rather uniform pinning/depinning forces, δ*F*, across the different binary mixture concentrations, with most average values self-contained within the standard deviations, are reasonable as per the larger changes in contact angle δ*θ* observed in the case of high ethanol concentration binary mixtures and pure ethanol. Since the increase in the ethanol concentration in turn decreases the fluid surface tension (see eq 1), consequently, greater changes in contact angle δ*θ* are required for a jump to ensue as earlier reported in Figure 6. This supports the larger distance of the jumps of the contact line in the case of pure ethanol when compared to the expected jump distance *s* + *d* for short pillar spacing (*s* ≤ 20 μm), as well as the larger jumps of the contact line for high ethanol concentrations when compared to high water concentrations for large pillar spacing (*s* ≥ 40 μm). For the same micropillared configuration, pure water and high surface tension binary mixtures require short jumps of the contact line to overcome similar pinning/depinning forces. The short magnitude of the jumps, in turn, gives rise to a larger number of discrete jumps, with the maximum number of these discrete jumps taking place for pure water and high water concentration fluids on the shortests micropillar spacing of *s* = 5 μm.

### Unified Discussion on Evaporation Mechanisms

**Figure 8:**
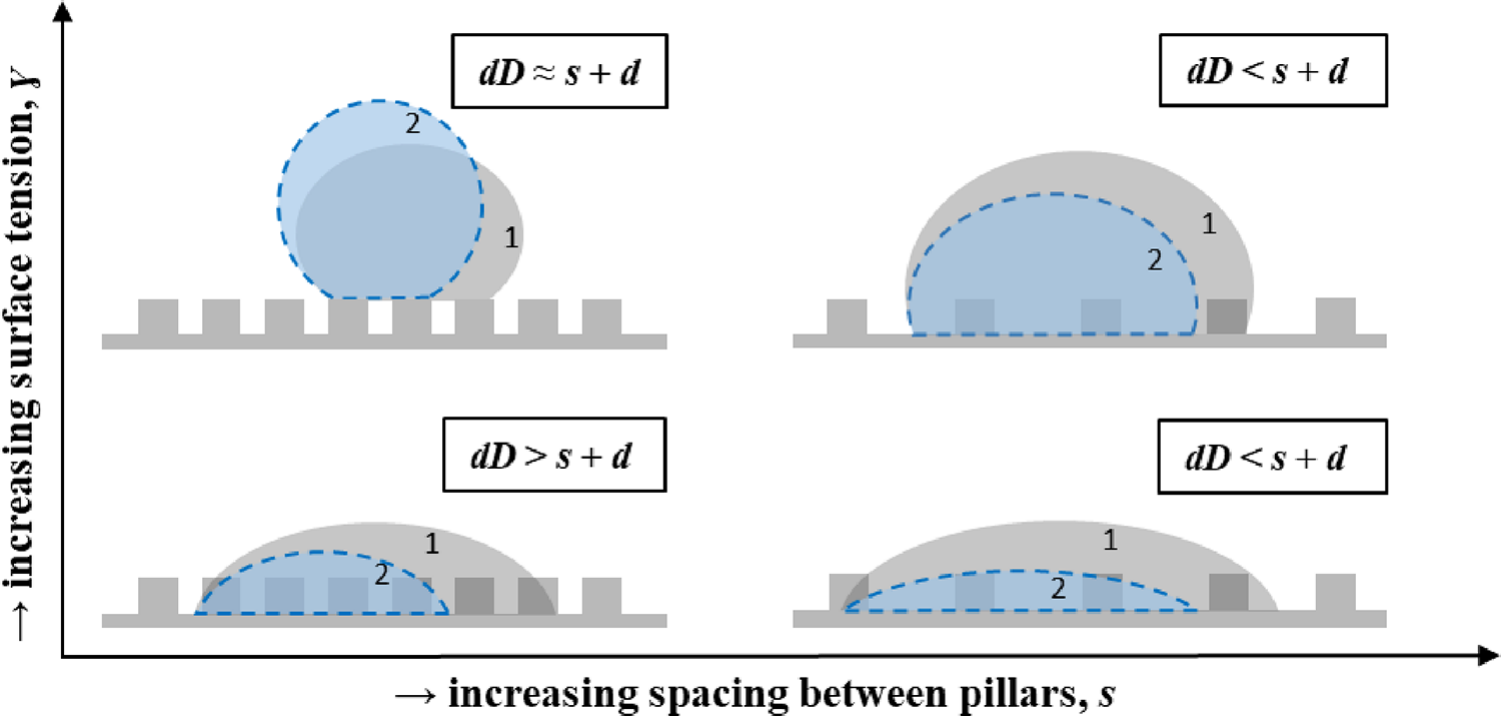
Schematic representation of the typical expected magnitude of the contact line jump, δ*D*, with respect to the expected jump of the contact line defined as *s* + *d*, function of the fluid surface tension, and micropillar spacing.

Based on the different results and analysis presented above, we propose the following unified typical evaporation mechanism function of the fluid surface tension and the pillar spacing, as shown in the schematics in Figure 8.

Figure 8 summarizes the different evaporation behaviors reported and a qualitative comparison of the reported magnitudes of the jumps with respect to the expected jump of the contact line or *s* + *d* during the stick–slip and/or the mixed stick–slip modes. On one hand, for high surface tension fluids, *i.e.*, pure water and its high water concentration mixtures, on short micropillar spacing, droplets typically sit in the Cassie–Baxter regime, and the jumps of the contact line ensue from one micropillar structure top to the next micropillar structure top, *i.e.*, δ*D* ≈ *s* + *d*. In comparison, for larger pillar spacing, there is no direct correlation between the magnitude of the jumps and the expected jump, although δ*D* is typically smaller than *s* + *d* and the droplets evaporate in a similar manner to the smooth counterpart with δ*D* < *s* + *d*.

On the other hand, as the ethanol concentration increases on short pillar spacing, droplets display a larger droplet footprint and lower contact angles in the partial non-wetting Wenzel regime with contact line jumps typically larger than the expected ones, *i.e.*, δ*D* > *s* + *d*. We note here that in these particular cases, the occurrence of asymetric droplets may lead to further deviation from the spherical cap with the consequent higher energy gained by the droplet and the reported greater jumps of the contact line. However, for these low surface tension fluids on large spacing, there is also no direct correlation between the magnitude of the jumps and the expected jump δ*D* < *s* + *d*, similar to the behavior reported for higher surface tension fluids.

We would like to further note here that in the case of binary mixtures, spreading or contraction of the droplet contact line may ensue depending on the volatility and the mixture as well as depending on the difference in surface tensions between the two miscible fluids, which generates a surface tension gradient locally near the contact line. In the presence of binary mixtures, solutal Marangoni induces further droplet spreading than their respective pure fluids, whereas if the most volatile component has the highest surface tension, then the retraction of the contact line may ensue.^26,44,45^ However, while this phenomena may occur on smooth surfaces, in the case of our structured surfaces, the presence of finite pinning sites hinders any further spreading or retracting dynamics of the contact line that may occur as a consequence of any surface tension gradient. This is further supported by the absence of any initial contact line spreading or retracting during the droplet evolution over time represented in Figure 3.

Local and overall pinning force and free energy analyses applied to the different evaporating phases, fluid concentration, and pillar structure, are proposed as further research aiming to reach a more holistic understanding of the discrete pinning and depinning mechanisms and energy barriers present during binary mixture sessile droplet evaporation on structured surfaces.

Last, we anticipate that the different new mixed stick–slip modes reported here are to be found for other evaporating fluids. More in particular, on one hand, the decreasing contact angle mixed stick–slip mode is to be found at the end of evaporation not only in the case of this or other binary mixture fluids but also in the case of pure fluids such as pure water at the end of the evaporation (Figure 3b,c) and for pure ethanol both in the middle and at the end of the evaporation (Figure 3j–l). On the other hand, for the increasing contact angle mixed stick–slip mode to ensue, the following condition must apply, which is that a noticeable contrast in surface tensions between the most volatile fluid having lower surface tension and the least volatile fluid having the higher surface tension must occur. This presumably excludes the occurrence of the increasing contact angle mixed stick–slip mode for binary mixtures of two highly volatile fluids with similar surface tension at early or medium times from the onset of evaporation, while its occurrence at the end of the evaporation still be possible as noticed for a pure volatile fluid such as ethanol due to the absorption-adsorption and/or condensation of water vapor onto the evaporating volatile fluid droplet.^12,13^

## Conclusions

The evaporation behavior of pure water and pure ethanol and their binary mixtures on hydrophobic structured surfaces has been experimentally investigated. Three different evaporative behaviors have been noticed: pinning, stick–slip, and mixed mode, with the absence of a constant contact angle mode, which are consistent with the literature. In addition, two further mixed stick–slip modes are reported here for the first time, namely, the increasing contact angle mixed stick–slip and the decreasing contact angle mixed stick–slip modes. The increasing contact angle mixed stick–slip mode occurs relatively near the beginning of the evaporation as the more volatile fluid, *i.e.*, ethanol, evaporates preferentially, and the water concentration within the droplet increases, which eventually increases locally the droplet surface tension leading to the contact angle increase upon a depinning event. This regime was also noticed for pure ethanol as water present in a humid environment adsorbs-absorbs and/or condenses on the droplet as ethanol evaporates. The decreasing contact angle mixed stick–slip mode occurs at the end of the droplet evaporation when the pillars affect considerably the contact line motion and the shape displayed by small volume droplets. The extent and duration of these evaporation modes are dependent on the fluid surface tension and on the pillar spacing, as reported here. Moreover, the magnitude of the stick–slip contact line jumps along with the changes in contact angle was studied. It was found that the contact line movement is dependent on the spacing between pillars and self-contained within the spacing and diameter of the pillared configuration (*s* + *d*) in the case of high surface tension fluids and short pillar spacing. For larger pillar spacing, the magnitude of the jumps is below the expected value *s* + *d*. In addition, all fluids used on structured surfaces exhibit similar values of pinning/depinning force except for short spacing where this force increases as the ethanol concentration increases. The increase in the pinning force for medium and high ethanol concentrations lies in the different wetting behaviors as partial non-wetting Wenzel and the consequent greater droplet–surface interactions when compared to Cassie–Baxter droplets, this latter regime ensuing in the case of low ethanol concentration and pure water. In comparison for large spacing, most/all average values are within the standard deviations. It is concluded that by choosing the appropriate surface structure and fluid surface tension, the wetting regime, droplet shape, initial pinning time, evaporation mode, and its duration can be tailored, which can prove to be beneficial to many engineering, biological, and/or medical applications.

## Supporting Information Available

Evolution of the contact angle and contact radius on all structured surfaces (Section S1) for pure water (Figure S1), 20% ethanol to 80% water (Figure S2), 40% ethanol to 60% water (Figure S3), 60% ethanol to 40% water (Figure S4), 80% ethanol to 20% water (Figure S5), and pure ethanol (Figure S6); stick–slip contact line (CL) jumps (Figure S7) and contact angle changes (Figure S8) including ethanol (E) preferential evaporation, pure water (W) evaporation, and the binary mixture (M) evaporation (Section S2); pinning/depinning force δ*F* (Figure S9) including ethanol (E) preferential evaporation, pure water (W) evaporation, and the binary mixture (M) evaporation (Section S3) (PDF)
