# Supplementary material for: Binary Mixture Droplet Evaporation on Microstructured Decorated Surfaces and the Mixed Stick–Slip Modes
Source: Langmuir. 2023 Jun 5;39(23):8323–38. doi: 10.1021/acs.langmuir.3c00914 (PMC10269432; doi:10.1021/acs.langmuir.3c00914)
Supplement: Supplementary file 1 — la3c00914_si_001.pdf [file la3c00914_si_001.pdf]

Supporting Information – Binary Mixture Droplet Evaporation on Micro-Structured Decorated Surfaces and the Mixed-Stick-Slip Modes

Khaloud Moosa Al Balushi[1,2], Gail Duursma[1], Prashant Valluri[1], Khellil Sefiane[1], Daniel Orejon[1,3*]

[1]Institute for Multiscale Thermofluids, School of Engineering, The University of Edinburgh, Edinburgh EH9 3FD, Scotland, UK

[2] Department of Engineering, The University of Technology and Applied Sciences, Suhar 311, Oman

[3] International Institute for Carbon-Neutral Energy Research (WPI-I2CNER), Kyushu University, 744 Motooka, Nishi-ku, Fukuoka 819-0395, Japan

* Corresponding author: d.orejon@ed.ac.uk

Abstract

The interactions between liquid droplets and solid surfaces during wetting and phase change are important to many applications and are related to the physicochemical properties of the substrate and the fluid. In this work, we investigate experimentally the evaporation pure water, pure ethanol and their binary mixture droplets, accessing a wide range of surface tensions, on hydrophobic micro-pillared surfaces varying the spacing between the pillars. Results show that on structured surfaces droplets evaporate following three classical evaporative behaviours: constant contact radius (CCR)/pinning, stick-slip, or mixed mode. In addition, we report two further evaporation modes which are a *mixed-stick-slip* mode where the contact angle increases whilst the contact radius decreases in a stick-slip fashion and a *mixed-stick-slip* mode where the contact angle and the contact radius decrease in a stick-slip fashion. We name these new evaporation modes as the *increasing* and *decreasing contact angle mixed-stick-slip* modes respectively. The former ensues since the fluid surface tensions increases as the most volatile fluid evaporates coupled to the presence of structures, whereas the latter is owed to the presence of structures for either fluid. The duration of each evaporation mode is dissimilar and depends on the surface tension and on the spacing between structures. Pure water yields longer initial pinning times on all surfaces before stick-slip ensues whereas for binary mixtures and pure ethanol, initial pinning ensues mainly on short spacing structures owed to the different wetting regimes displayed. Whereas high ethanol concentration binary mixtures droplets evaporate following the stick-slip mode for longer periods of time independent of the solid fraction. *Mixed-stick-slip* modes ensue mainly for high ethanol concentrations and/or pure ethanol on short spacing structures. This investigation provides guidelines for tailoring the evaporation of a wide range of surface tension fluids on structured surfaces for inkjet printing, DNA patterning or microfluidics applications.

Keywords: Evaporation, Binary mixtures, Micro-decorated surfaces, Pinning, Stick-slip, Mixed-stick-slip mode

SI.1 – Evolution of the contact angle and contact radius

Figures SI-1 – SI-6 represent the average evolution of the (dark green) contact angle, θ (deg), and (dark red) contact radius, R (mm), for all fluids used in this study and on all surfaces used for at least three independent experiments. The shaded area illustrates the standard deviation. Evaporative behaviour on the smooth hydrophobic counterpart is included for comparison along with the results for 160 µm spacing. Stick-slip modes are identified by analysing the data for a change in the diameter, $0.005 < dD < 0.02$.

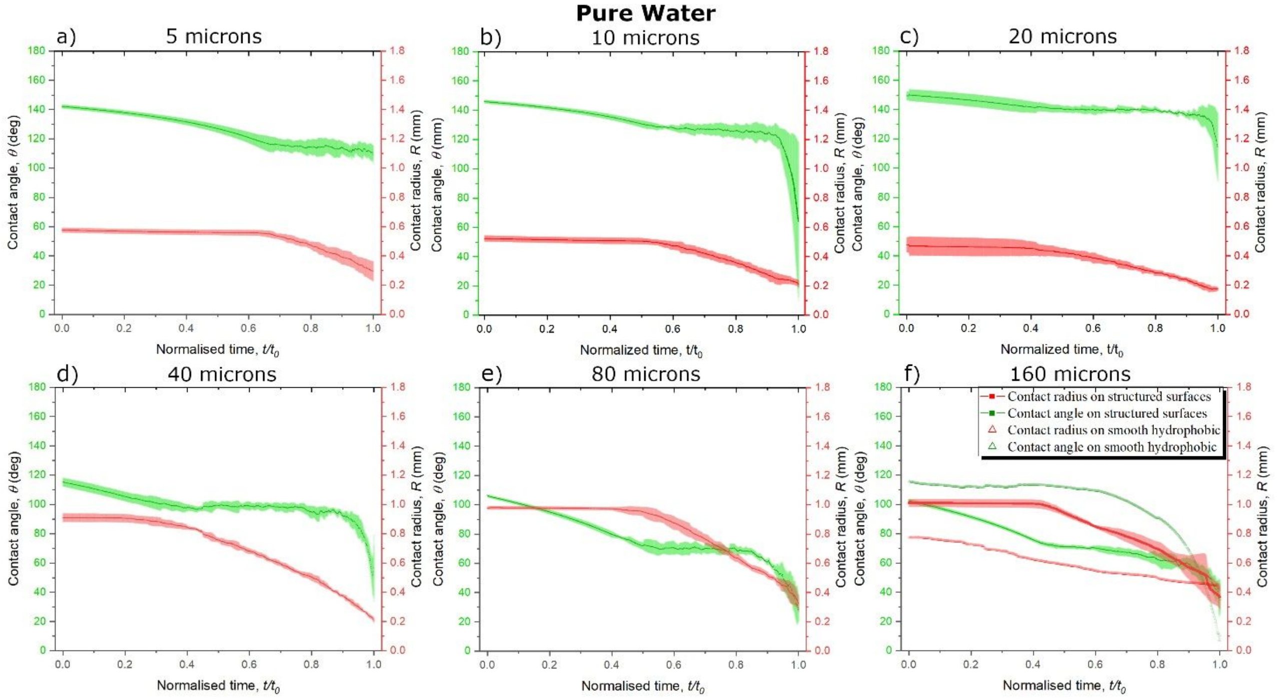

Figure SI-1. The solid line represents the average evolution of the (dark green) contact angle, θ (deg), and (dark red) contact radius, R (mm), for pure water on a) 5 µm spacing, b) 10 µm spacing, c) 20 µm spacing, d) 40 µm spacing, e) 80 µm spacing, f) 160 µm spacing, of at least three independent experiments, while shaded area illustrates the standard deviation. Evaporative behaviour on the smooth hydrophobic counterpart is included for comparison along with the results for 160 µm spacing.

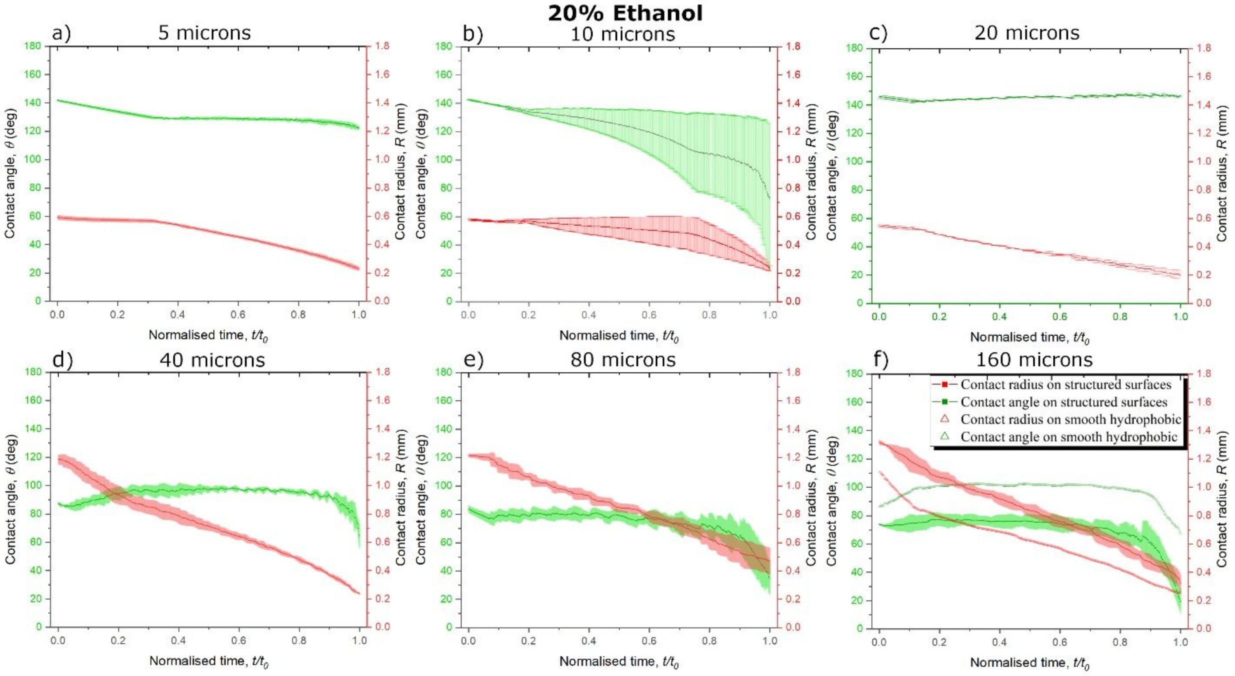

Figure SI-2. The solid line represents the average evolution of the (dark green) contact angle, θ (deg), and (dark red) contact radius, R (mm), for 20% ethanol on a) 5 µm spacing, b) 10 µm spacing, c) 20 µm spacing, d) 40 µm spacing, e) 80 µm spacing, f) 160 µm spacing, of at least three independent experiments, while shaded area illustrates the standard deviation. Evaporative behaviour on the smooth hydrophobic counterpart is included for comparison along with the results for 160 µm spacing.

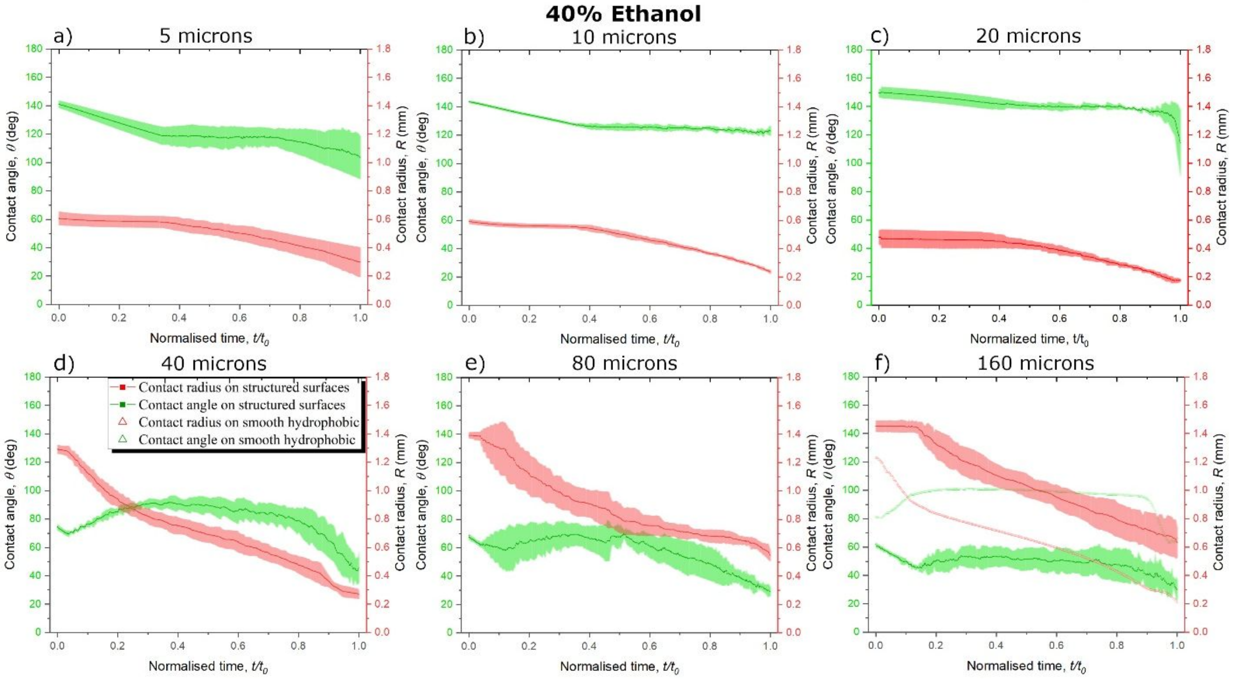

Figure SI-3. The solid line represents the average evolution of the (dark green) contact angle, θ (deg), and (dark red) contact radius, R (mm), for 40% ethanol on a) 5 µm spacing, b) 10 µm spacing, c) 20 µm spacing, d) 40 µm spacing, e) 80 µm spacing, f) 160 µm spacing, of at least three independent experiments, while shaded area illustrates the standard deviation. Evaporative behaviour on the smooth hydrophobic counterpart is included for comparison along with the results for 160 µm spacing.

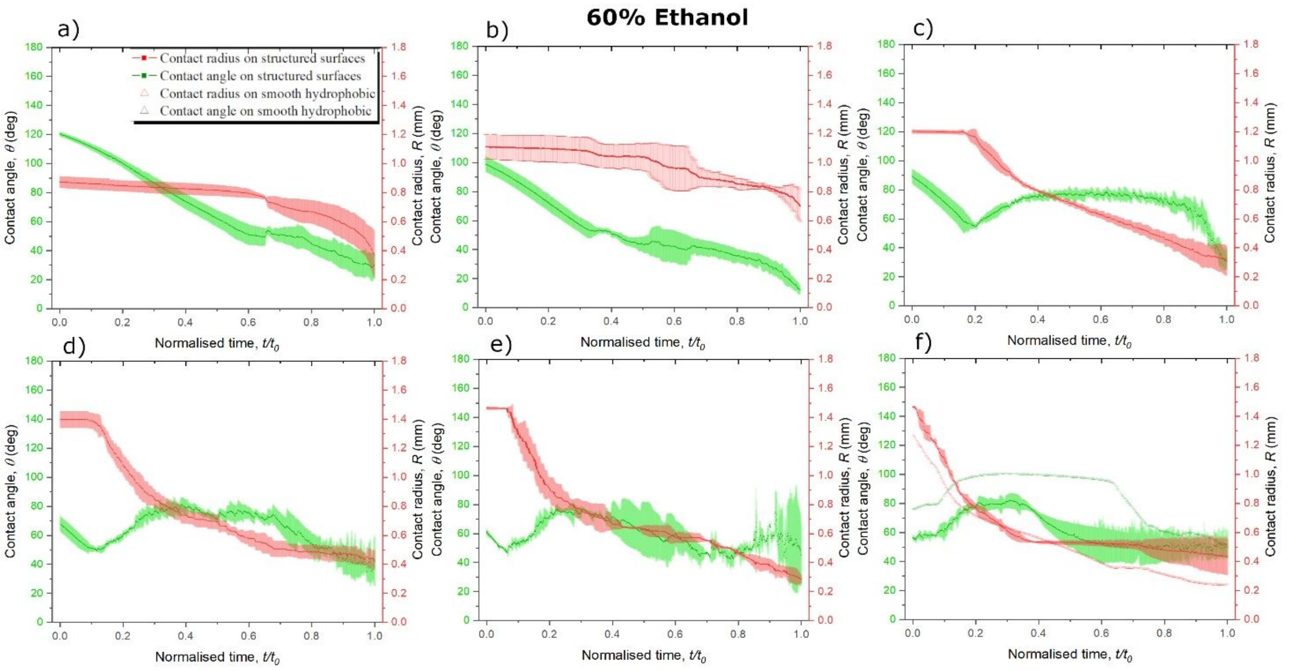

Figure SI-4. The solid line represents the average evolution of the (dark green) contact angle, θ (deg), and (dark red) contact radius, R (mm), for 60% ethanol on a) 5 µm spacing, b) 10 µm spacing, c) 20 µm spacing, d) 40 µm spacing, e) 80 µm spacing, f) 160 µm spacing, of at least three independent experiments, while shaded area illustrates the standard deviation. Evaporative behaviour on the smooth hydrophobic counterpart is included for comparison along with the results for 160 µm spacing.

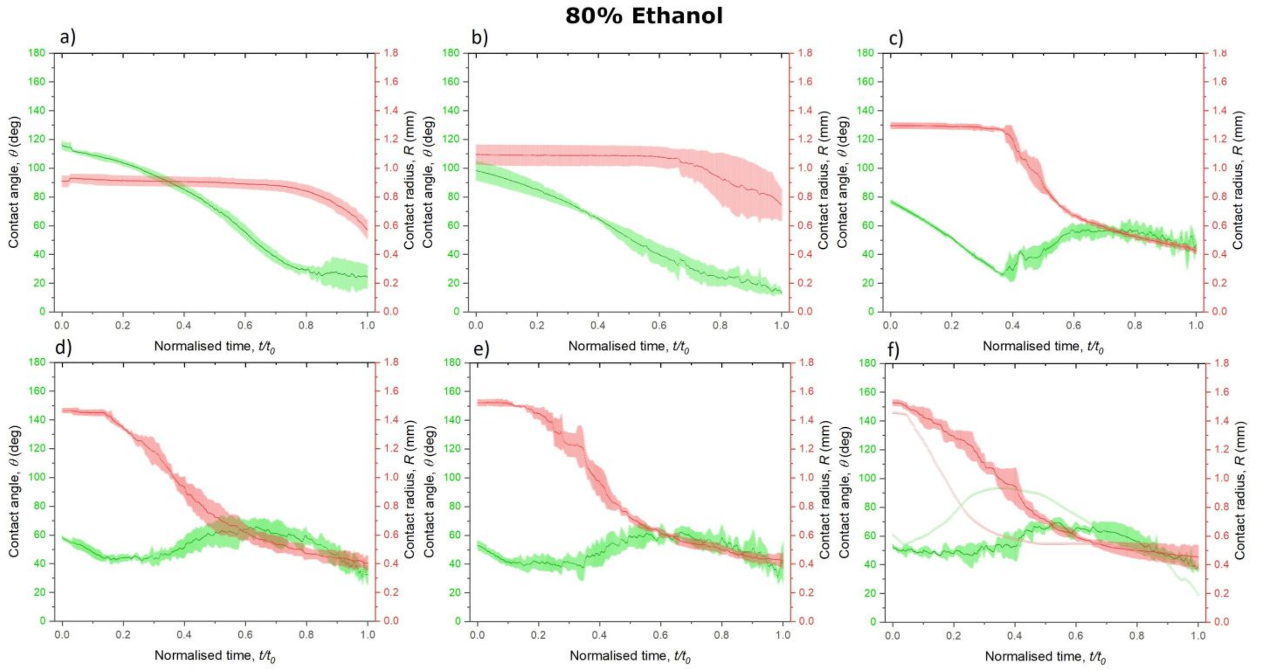

Figure SI-5. The solid line represents the average evolution of the (dark green) contact angle, θ (deg), and (dark red) contact radius, R (mm), for 80% ethanol on a) 5 µm spacing, b) 10 µm spacing, c) 20 µm spacing, d) 40 µm spacing, e) 80 µm spacing, f) 160 µm spacing, of at least three independent experiments, while shaded area illustrates the standard deviation. Evaporative behaviour on the smooth hydrophobic counterpart is included for comparison along with the results for 160 µm spacing.

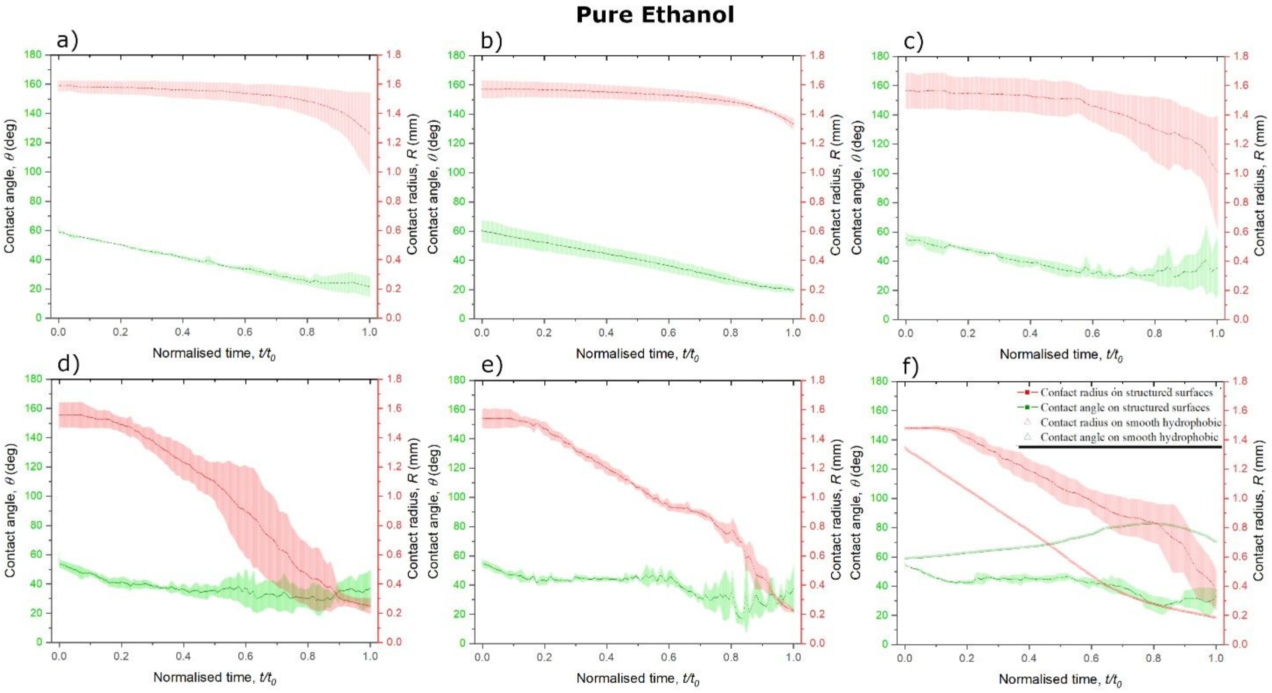

Figure SI-6. The solid line represents the average evolution of the (dark green) contact angle, θ (deg), and (dark red) contact radius, R (mm), for 80% ethanol on a) 5 µm spacing, b) 10 µm spacing, c) 20 µm spacing, d) 40 µm spacing, e) 80 µm spacing, f) 160 µm spacing, of at least three independent experiments, while shaded area illustrates the standard deviation. Evaporative behaviour on the smooth hydrophobic counterpart is included for comparison along with the results for 160 µm spacing.

SI.2 – Stick-slip contact line (CL) jumps and contact angle changes

Figures SI-7 and SI-8 show and compare the average contact line jumps, δD, and the average changes in the contact angle, $\delta\theta$, respectively, for the different fluids used and the different structured surfaces investigated. The magnitudes of contact line jump and contact angle changes are compared between ethanol and pure water evaporation and their mixtures. Black columns (E) represent (phase I) assuming ethanol evaporation typically occurs at the first stage of the binary mixture evaporation, blue columns (W) represent (phase II) pure water evaporation ensuing after most/all ethanol has evaporated, and red columns (M) represent the average values over the complete binary mixture evaporation process without distinguishing the evaporating component, i.e., ethanol and water evaporating.

For larger spacing $s \geq 40$ μm, no clear trends are seen, but in the case of the binary mixtures, the magnitude of the change in contact angle during phase II, i.e., water evaporation, is larger than that estimated for the overall evaporation, which is explained in terms of the smaller number of jumps taking place during pure ethanol evaporation in phase I.

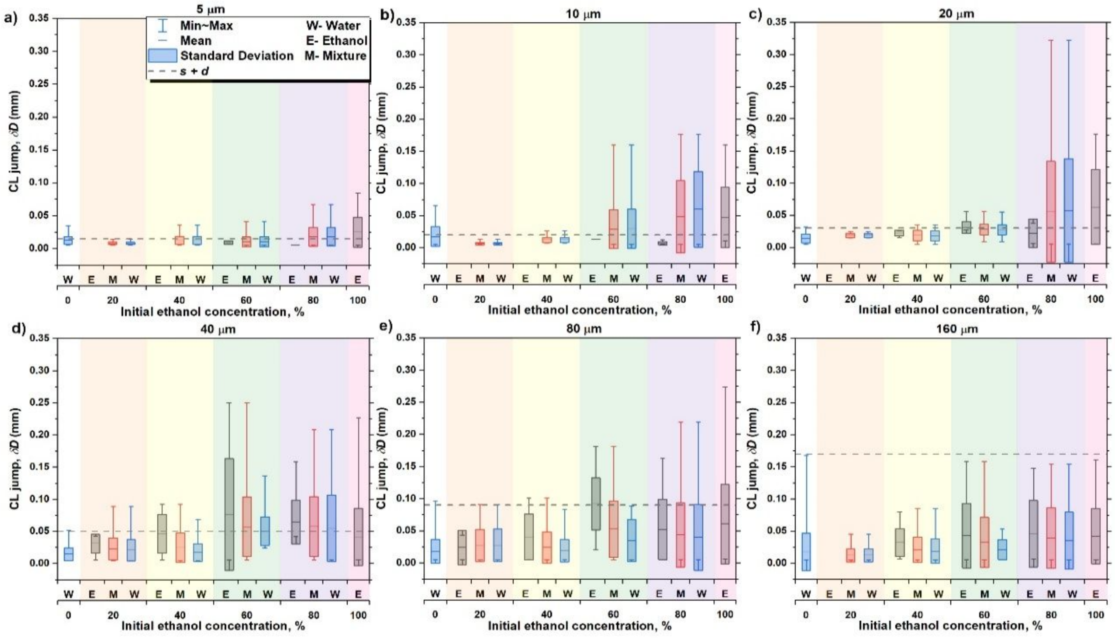

Figure SI-7. Average jump distance, δD (mm), of the contact line (CL) for the different ethanol concentrations on a) 5 μm, b) 10 μm, c) 20 μm, d) 40 μm, e) 80 μm, f) 160 μm pillar spacing, solid horizontal lines represent the average, the closed box columns represent the standard deviation, and whiskers represent the maximum and minimum value of the change in the contact line. Black columns represent pure ethanol (E) and phase I, blue columns represent pure water (W) and phase II, red columns represent the binary mixtures (M), and the grey dashed lines show the expected jump distance equals $s + d$. % indicates the initial ethanol concentration. The different shaded areas represent the six fluids used in this study. Note that the average jump distance, δD (mm), and standard deviation were calculated from the local jumps taking place for three independent experiments.

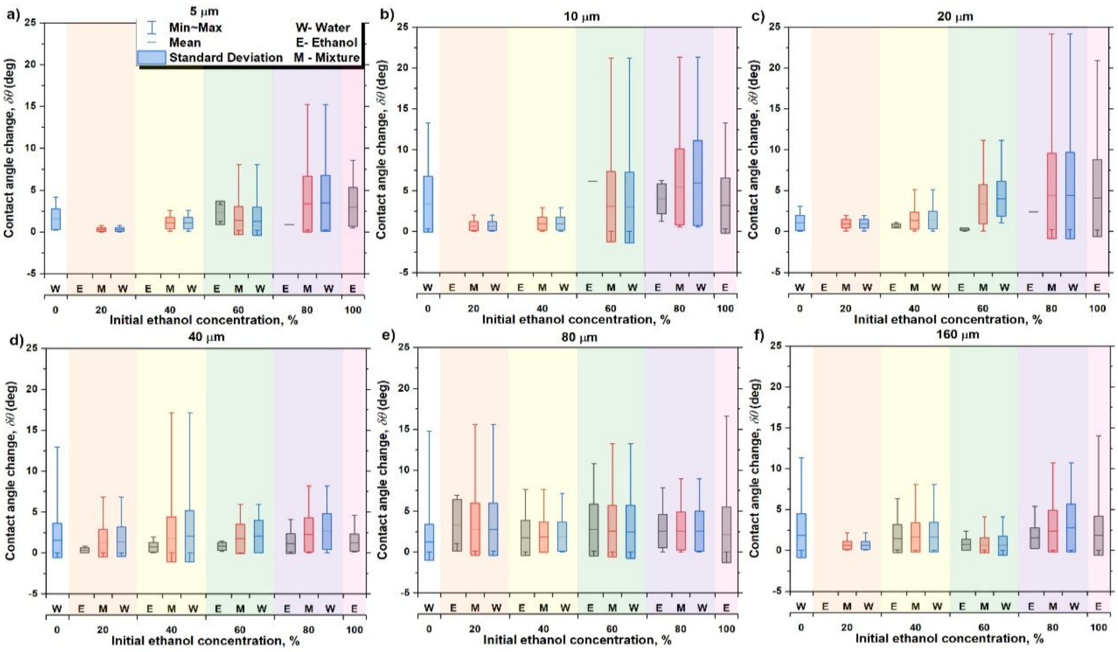

Figure SI-8. Average jump distance, $\delta\theta$ (mm), of the contact line (CL) for the different ethanol concentrations on a) 5 µm, b) 10 µm, c) 20 µm, d) 40 µm, e) 80 µm, f) 160 µm pillar spacing, solid horizontal lines represent the average, the closed box columns represent the standard deviation, and whiskers represent the maximum and minimum value of the change in the contact line. Black columns represent pure ethanol (E) and phase I, blue columns represent pure water (W) and phase II, red columns represent the binary mixtures (M), and the grey dashed lines show the expected jump distance equals $s + d$. % indicates the initial ethanol concentration. The different shaded areas represent the six fluids used in this study. Note that the average jump distance, δ*D* (mm), and standard deviation were calculated from the local jumps taking place for three independent experiments.

SI.3 – Pinning/ depinning force δF

Figures SI-9 shows and compares the average absolute pinning force, δF, for the different fluids used and the different structured surfaces investigated. The magnitudes of the δF are compared between ethanol and pure water evaporation and their mixtures. Black columns (E) represent (phase I) assuming ethanol evaporation typically occurs at the first stage of the binary mixture evaporation, blue columns (W) represent (phase II) pure water evaporation ensuing after most/all ethanol has evaporated, and red columns (M) represent the average values over the complete binary mixture evaporation process without distinguishing the evaporating component, i.e., ethanol and water evaporating.

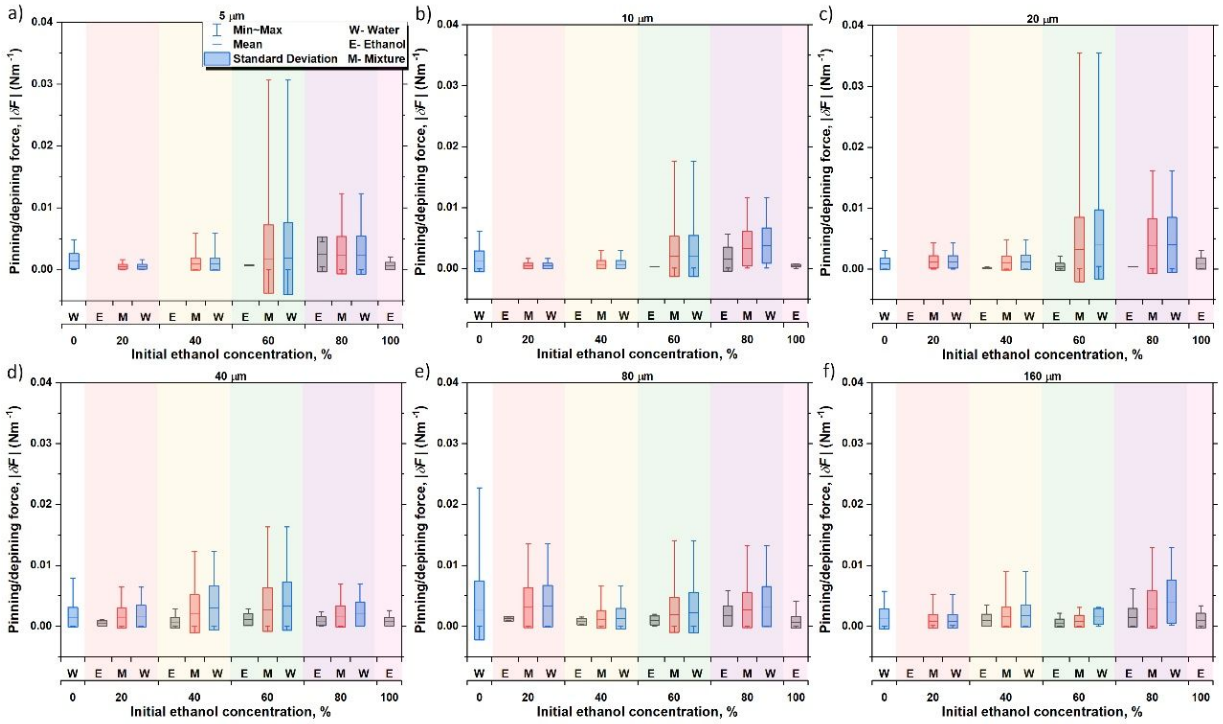

Figure SI-9 Average pinning/depinning force, δF (N/m), for the different pure fluids and binary mixtures on a) 5 µm, b) 10 µm, c) 20 µm, d) 40 µm, e) 80 µm, f) 160 µm. Solid horizontal lines represent the average, the closed box columns represent the standard deviation, and whiskers represent the maximum and minimum value of the change in the pinning/depinning force. Black columns represent pure ethanol (E) and phase I, blue columns represent pure water (W) and phase II, red columns represent the binary mixtures (M). The different background shaded areas represent the six fluids used in this study. Note that the average pinning/depinning force, δF (N/m), and standard deviation were calculated from the local jumps taking place for three independent experiments.
